# Cognitive Development and Brain Gray Matter Susceptibility to Prenatal Adversities: Moderation by the Prefrontal Cortex Brain-Derived Neurotrophic Factor Gene Co-expression Network

**DOI:** 10.3389/fnins.2021.744743

**Published:** 2021-11-24

**Authors:** Euclides José de Mendonça Filho, Barbara Barth, Denise Ruschel Bandeira, Randriely Merscher Sobreira de Lima, Danusa Mar Arcego, Carla Dalmaz, Irina Pokhvisneva, Roberto Britto Sassi, Geoffrey B. C. Hall, Michael J. Meaney, Patricia Pelufo Silveira

**Affiliations:** ^1^Department of Psychiatry, McGill University, Montreal, QC, Canada; ^2^Ludmer Centre for Neuroinformatics and Mental Health, Douglas Hospital Research Center, Montreal, QC, Canada; ^3^Integrated Program in Neuroscience, Faculty of Medicine, McGill University, Montreal, QC, Canada; ^4^Programa de Pós-Graduação em Psicologia, Universidade Federal do Rio Grande do Sul, Porto Alegre, Brazil; ^5^Programa de Pós-Graduação em Bioquímica e Neurociências, Universidade Federal do Rio Grande do Sul, Porto Alegre, Brazil; ^6^Department of Psychiatry, University of British Columbia, Vancouver, BC, Canada; ^7^Department of Psychology, Neuroscience & Behaviour, McMaster University, Hamilton, ON, Canada; ^8^Singapore Institute for Clinical Sciences, Agency for Science, Technology and Research (A*STAR), Singapore, Singapore

**Keywords:** BDNF, polygenic score, prenatal adversity, cognitive development, gray matter

## Abstract

**Background:** Previous studies focused on the relationship between prenatal conditions and neurodevelopmental outcomes later in life, but few have explored the interplay between gene co-expression networks and prenatal adversity conditions on cognitive development trajectories and gray matter density.

**Methods:** We analyzed the moderation effects of an expression polygenic score (ePRS) for the Brain-derived Neurotrophic Factor gene network (BDNF ePRS) on the association between prenatal adversity and child cognitive development. A score based on genes co-expressed with the prefrontal cortex (PFC) BDNF was created, using the effect size of the association between the individual single nucleotide polymorphisms (SNP) and the BDNF expression in the PFC. Cognitive development trajectories of 157 young children from the Maternal Adversity, Vulnerability and Neurodevelopment (MAVAN) cohort were assessed longitudinally in 4-time points (6, 12, 18, and 36 months) using the Bayley-II mental scales.

**Results:** Linear mixed-effects modeling indicated that BDNF ePRS moderates the effects of prenatal adversity on cognitive growth. In children with high BDNF ePRS, higher prenatal adversity was associated with slower cognitive development in comparison with those exposed to lower prenatal adversity. Parallel-Independent Component Analysis (pICA) suggested that associations of expression-based SNPs and gray matter density significantly differed between low and high prenatal adversity groups. The brain IC included areas involved in visual association processes (Brodmann area 19 and 18), reallocation of attention, and integration of information across the supramodal cortex (Brodmann area 10).

**Conclusion:** Cognitive development trajectories and brain gray matter seem to be influenced by the interplay of prenatal environmental conditions and the expression of an important BDNF gene network that guides the growth and plasticity of neurons and synapses.

## Introduction

Brain-derived Neurotrophic Factor (BDNF) is a protein involved in several biological pathways – from neurogenesis, promotion of neuronal survival and differentiation, to modulation of synaptic plasticity – playing a central role in both the developing and adult nervous system (Hempstead, 2014). Acting through its high-affinity tyrosine receptor kinase B (TrkB) receptor, it mediates neurite and spine outgrowth (Binder and Scharfman, 2004; Ji et al., 2005), and this signaling is also important for synaptic plasticity (Ji et al., 2005; Lu et al., 2014), a phenomenon that enables the organism to change according to environmental stimuli, and makes possible learning and memory. Also, it controls short and long-lasting synaptic interactions in the hippocampus, and its expression mediates working memory processes in the prefrontal cortex (Gold et al., 2003; Xing et al., 2012; Kowiański et al., 2018). BDNF is expressed in almost all brain regions, but the highest levels are found in the frontal cortex, hippocampus, and amygdala (West et al., 2014). Several studies indicate altered BDNF expression in brain structures like the prefrontal cortex (PFC), hippocampus, and striatum in post-mortem human brains of patients that suffered psychiatric illnesses. There are decreased levels of BDNF mRNA and protein expression in the hippocampus of suicide victims (Banerjee et al., 2013), and significant differences in BDNF transcripts allow to distinguish schizophrenia, bipolar disorder, and major depressive disorder patients from healthy subjects, suggesting that the BDNF system is implicated in several physiological aspects of brain development (Molendijk et al., 2012; Banerjee et al., 2013; Reinhart et al., 2015).

Prenatal exposure to stress, maternal depression/anxiety, low social support, and poor access to prenatal health services have long-term effects on child cognitive development that are well documented (Monk et al., 2012; O’Donnell et al., 2014a; Silveira et al., 2017). Brain plasticity and maturation are affected by positive and negative environmental exposures during sensitive periods of development (Nelson and Gabard-Durnam, 2020). The brain matures in a hierarchical manner, meaning that the quality of maturation of early-developing regions will affect the subsequent development of other regions (Tottenham, 2019). Gene expression in different brain regions at different developmental stages indicates that timing is an important factor at the transcriptome level (Somel et al., 2009; Haeussler et al., 2017). This makes complex cerebral regions, for instance, the PFC, particularly sensitive to environmental conditions. The PCF receives several inputs from all other cortical areas, playing a key role in planning and performance of higher thinking, cognitive, affective, and social behaviors throughout development (Kolb et al., 2012); such interconnectivity results in a longer period needed for maturation (Fuster, 2015).

Expression of BDNF, and TrkB receptors begins early during brain development, especially in the cortical plate, both in rodents and primates (for a review, see Bartkowska et al., 2010). Therefore, it is not surprising that disturbances in its function early in life have remarkable effects upon neuronal structure and function. For example, transgenic mice with a functional reduction in BDNF or TrkB genes have a curtailment of dendritic arborization in cortical neurons in the prepubertal period (Xu et al., 2000; Gorski et al., 2003), and impairments in memory (Gorski et al., 2003). In this scenario, studies using animal models of prenatal stress have reported altered BDNF signaling during post-natal development (Badihian et al., 2020; Sobolewski et al., 2020). Stressors such as maternal immune activation during gestation, repeated restraint, or variable stress during pregnancy, cause altered BDNF expression in the PFC at different ages during development of the offspring (Matrisciano et al., 2012; Hemmerle et al., 2015; Niu et al., 2020). Accordingly, prefrontal TrkB and glucocorticoid receptor (GR) activities are known to be modulated by exposure to stressors (reviewed in Barfield and Gourley, 2018), and TrkB-GR interaction has been suggested (Numakawa et al., 2009). Therefore, prolonged variations in glucocorticoids could affect both GR and BDNF-TrkB function in the PFC (Barfield and Gourley, 2018), contributing to stress-induced cognitive alterations. In addition, abnormal signaling in the BDNF/TrkB pathway was reported to lead to abnormalities in the GABAergic and glutamatergic activities in the PFC (Sakata et al., 2009; Zhang et al., 2013).

Negative exposures during the prenatal and early postnatal period have been associated with cognitive and brain development in different ways. Behaviorally, the attainment of cognitive skills is understood as a developmental cascade, characterized by a cumulative process in which functioning at a lower level of behavior (e.g., visuomotor integration, fine motor skills, habituation) affects higher-level functions that develop later (e.g., IQ, language and executive functions) (Almas et al., 2016; Choi et al., 2018; Camerota and Willoughby, 2019). In terms of neurodevelopment, experiments with infant rats exposed to caretakers that displayed abusive behaviors show increased levels of methylation of BDNF DNA throughout the life span, and reduced BDNF gene expression in the adult PFC (Roth et al., 2009). Prenatal exposure to stress was also associated with high methylation and lower expression of the BDNF gene in the PFC and hippocampus (Roth et al., 2011; Badihian et al., 2020). In humans, brain structure is also impacted by early life stressors, resulting in several morphological and functional alterations (Buss et al., 2010; Hair et al., 2015; Noble et al., 2015). The mentioned interrelated pathways affect the developing individual resulting in a predisposition for disease and poorer developmental outcomes later in life. Adolescence is also a sensitive period for PFC development. The PFC is one of the last brain regions to mature (Fuster, 2015; Hoops and Flores, 2017), and it is known to undergo significant structural remodeling, with dendritic and synaptic pruning during adolescence (Bourgeois et al., 1994; Shaw et al., 2020). This period of synaptic remodeling is believed to generate a refinement of connections (Barfield and Gourley, 2018). Therefore, exposure to adversities during this period can impact on PFC circuitry, and on adult behavior (Shaw et al., 2020). However, before this period of pruning, there is an initial phase of neuronal differentiation, dendritic spine and synapse overproduction, that occurs during prenatal and early childhood periods that will influence future development, stressing the relance of this sensitive window (Bourgeois et al., 1994; Lotfipour et al., 2009).

In summary, a large body of evidence indicates that early exposure to environmental adversity affects cognitive development, and some individuals are more susceptible than others to this long-term effect. Individual differences likely affect the impact of environmental exposure on several child developmental outcomes (Belsky, 2013; Silveira et al., 2017). It was shown that genetic variation in the BDNF gene (the Val66Met polymorphism), which decreases BDNF function (Egan et al., 2003), can lead to lower memory levels (Egan et al., 2003), and is associated with impairment in executive functioning (Nagata et al., 2012). This is particularly significant in individuals with high levels of early life adversity (Gabrys et al., 2017), in which this variation was associated with difficulties in attentional flexibility, a PFC-based function. Also, previous studies involving Val66Met polymorphisms suggested a role of the BDNF gene in moderating the effects of early adversity on attention problems and child behavior (Drury et al., 2012; Gunnar et al., 2012; O’Donnell et al., 2014b). However, it is known that the action of a gene is not isolated, but correlated in concert with other genes in functional networks (Gaiteri et al., 2014).

Genome-wide association studies (GWAS) are an important technological advance for the understanding of human health and disease but are still not able to inform the underlying tissue-specific mechanisms that explain phenotypic variation (Silveira et al., 2017; Hari Dass et al., 2019). GWAS considers only the highly significant genetic variants associated with a disease, thus are not enlightening of the several manifestations or endophenotypes that may precede the phenotype (Dalle Molle et al., 2017; Hari Dass et al., 2019). We propose a novel genomics approach, using a biologically informed genetic score based on genes co-expressed with the BDNF gene specifically in the PFC (BDNF ePRS) during the prenatal and early life periods to investigate the association with child cognitive development from 6 to 36 months of age. For a sub-sample of participants that we were able to follow up and collect structural Magnetic resonance images at age 9 we analyzed the multivariate association between the single nucleotide polymorphisms (SNPs) from the BDNF ePRS and gray matter density in order to uncover the mechanism of the interaction between prenatal environment and genotype and its association with brain development.

## Materials and Methods

### Participants and Cohort Characteristics

Participants’ data were derived from the Maternal Adversity, Vulnerability and Neurodevelopment prospective community-based cohort MAVAN (O’Donnell et al., 2014). A hundred and fifty-seven children from two sites - Montreal (Québec) and Hamilton (Ontario), Canada - composed the sample of the present study. Pregnant women were recruited around 13 to 20 weeks of gestation from obstetric clinics in hospitals. They were eligible to take part in the study if over 18 years of age, fluent in either English or French, and did not have serious obstetric complications during the pregnancy or delivery of the child, had a child with extremely low birth weight, or had any congenital diseases. Children were monitored from birth up to 12 years of age using several assessments of neurodevelopment. Ethical approval for this study was obtained from the Douglas Mental Health University Institute (Montreal) and St-Joseph’s Healthcare (Hamilton Integrated Research Ethics Board). For this work, we considered cognitive neurodevelopmental data from the 6, 12, 18, and 36-months postnatal periods (*N* = 157), and a magnetic resonance imaging from a follow-up sample of 47 children at age nine (mean age = 9.3, *SD* = 1.4), the characteristics of the sample are shown in Table 1.

**TABLE 1:** Maternal Adversity, Vulnerability and Neurodevelopment (MAVAN) sample characteristics.

Variables	Cognitive development sample (6–36 months)	MRI sample (9 years)
	
	*N* = 157	*N* = 47
Gestational weeks, *M* (SD)	39.0 (1.2)	39.3 (1.2)
Birth weight (grams), *M* (SD)	3326.3 (448.3)	3256.1(458.7)
Income less than $30,000 a year	26 (16.5%)	16 (34.0%)
Maternal education: some community-college or less	14 (8.9%)	6 (12.8%)
Male sex	76 (48.4%)	28 (59.6%)
Smoking during pregnancy	17 (10.8%)	11 (23.4%)
Montreal site	81 (51.5%)	38 (80.8%)
Cumulative prenatal score, *M* (SD)	1.3 (1.2)	1.2 (1.2)

### Measures

#### Cumulative Prenatal Adversity Score

The cumulative prenatal adversity score is a measure used to describe prenatal adversity conditions. It is composed of several indicators identified in the literature as being related to negative children’s outcomes (Silveira et al., 2017). It surveyed pregnancy conditions, maternal mental health during pregnancy (anxiety, depression), presence of chronic diseases such as diabetes, hypertension, vaginal spotting or bleeding, smoking during pregnancy, low birth size percentile, gestational age, and socioeconomic characteristics. Further descriptions of all instruments included in the cumulative prenatal adversity environment are presented in Table 2. For each met criterion - such as size percentile below 10th percentile or above 90th percentile or smoking during pregnancy - one point was given and all points were summed to obtain the adversity score. For psychometric scales, we considered 85th percentile as a cut-off value for positive screening stated by the instrument.

**TABLE 2:** Psychometric scales used to compose the Cumulative Prenatal Adversity Score.

Measure	Description	Scoring
Daily Hassles Scale (Lobel and Dunkel-schetter, 1990)	Indicates the level of struggle and frequency in respect to lack of money for basic needs such as food and electricity since the beginning of pregnancy. The mean test-retest reliability of the scale is.79.	Lack of money score above 9.
Center for Epidemiological Studies Depression Scale (CESD) (Radolf, 1977)	Assesses depressive symptomatology in the general population with emphasis on affective and somatic components. 20 items are scored on a 4-point Likert scale and high scores indicate more severe depressive symptoms. The internal consistency of the scale is.85 (coefficient Alpha).	Prenatal depression scores above 22.
State-Trait Anxiety Inventory (STAI) (Spielberger, 1989)	A measure of trait and state anxiety composed of 20 items for each construct. Internal consistency coefficients for the scales ranged from.86 to.95.	Pregnancy anxiety score above 1.95.
Abuse Assessment Screen (Newberger et al., 1992)	Presence of domestic violence or sexual abuse during pregnancy.	One point for the presence.
Marital Strain Scale (Pearlin and Schooler, 1978)	The Marital Strain Scale of Pearlin and Schooler is used to assess chronic stress with the romantic partner.	Marital strain score less than 2.9.
Health during pregnancy	Presence of chronic diseases during pregnancy: diabetes, hypertension, asthma, current or resolved), current severe vomiting, vaginal spotting or bleeding during the past 4–6 weeks, current anemia/constipation/blood in stool, or current vaginal/cervical/urinary tract infection/diarrhea.	One point for the occurrence of any pathology.
Smoking	Smoking anytime during pregnancy.	One point for the presence.
Gestational age	Gestational age in weeks.	One point if gestational age ≤ 37 weeks.
Birth size	Birth size percentile bellow 10th percentile or above 90th percentile	One point for the presence.
Income	Household total gross income.	One point if less than $30,000 a year.

#### Cognitive Development Measure

##### The Bayley Mental Scale of Infant Development

The Bayley Mental Scales (BSID-II) development index (MDI) is a composite of children’s language and cognitive abilities. It assesses age-appropriate levels of memory, problem-solving, habituation, incipient number concepts, generalization, classification, vocalizations, and language skills (Bayley, 1993). Psychometric properties of the Bayley scale indicated good to excellent evidence for the validity and reliability of the scale (Silva et al., 2020). Children’s development assessment was performed by trained and experienced professionals.

#### Brain-Derived Neurotrophic Factor Gene Network Score

##### Genotyping

At first, genetic variation in children was described using genome-wide platforms PsychChip and PsychArray (Illumina) using 200 ng of genomic DNA collected from buccal epithelial cells. SNPs with low call rate (bellow 95%), low *p*-values on Hardy-Weinberg Equilibrium exact test (*p* < 1e-40), and minor allele frequency smaller than 5% were removed. Quality control (QC) procedure was carried out using PLINK 1.951 (Purcell et al., 2007). Samples of individuals with a call rate less than 90% were also excluded. Imputation was performed using the Sanger Imputation Service and the Haplotype Reference Consortium (HRC) as the reference panel (release 1.1) by McCarthy et al. (2016) resulting in 20,790,893 SNPs with an information score higher than 0.80 and posterior genotype probabilities over 0.90.

##### Brain-Derived Neurotrophic Factor Expression Polygenic Score

The BDNF ePRS was calculated considering genes co-expressed with the BDNF gene in the PFC following the protocol described at Silveira et al. (2017) and Hari Dass et al. (2019). Three genetic databases were involved in thesis process: the *Genenetwork*^1^, *Brainspan*^2^, and *GTEx* (Genotype-Tissue Expression The GTEx Consortium, 2013^3^).

First, using the *Genenetwork*, the genes co-expressed with the BDNF gene in the PFC in mice were selected considering an absolute co-expression correlation equal to or higher than 0.5. Based on the Mouse Genome Informatics (MGI) database we identified human homologous genes. Then, we considered the *Brainspan* database to select human homologous transcripts that are enriched during the prenatal period to five years of age in the human PFC. At this point, we selected only transcripts that were differentially expressed in the PFC at ≥ 1.5-fold in comparison with adult samples, this list had 51 genes. This list was used to select individual SNPs within start/end ± 500 bp position of the genes according to NCBI in humans (the National Center for Biotechnology Information, United States National Library of Medicine^4^). From the gathered SNPs we retained only common SNPs between MAVAN genodata and GTEx and applied a linkage disequilibrium clumping procedure (*r*^2^ > 0.2), to keep independent SNPs with the lowest association p-values in the across 500 kb region. The final list consisted of 46 genes, with a 473 SNPs included in the BDNF ePRS.

Finally, to calculate the BDNF ePRS score we used the GTEx as a reference to weight the selected 473 SNPs. We multiplied the number of effect alleles for each SNP by the estimated coefficient of the association between each SNP and the genes’ expression in the PFC and by the sign of correlation between the gene expression of the particular gene and the BDNF. We summed all weighted SNPs to obtain the PFC BDNF ePRS score. High ePRS scores indicate higher predicted expression levels of genes that composed the BDNF network. The calculation of the BDNF ePRS score was done using PRSoS software tool (Chen et al., 2018).

In order to control for population stratification a principal component analysis was performed using SMARTPCA on the pruned dataset. For the pruned dataset we kept common variants (MAF > 0.05), not in linkage disequilibrium (*r*^2^ < 0.20, with a sliding window of 50 kb and an increment of 5 SNPs). Pruning was performed using PLINK 1.9. Based on a screen plot inspection the first three principal components that were the most informative of population structure were retained (Price et al., 2006). For validation of how the gene network scores change across brain regions, developmental stage, and gene of interest see Hari Dass et al. (2019).

### Data Analysis

#### Brain-Derived Neurotrophic Factor Expression Polygenic Score Enrichment Analysis

Biological interpretation of genes that comprised our genetic score was performed using enrichment analysis using MetaCore^TM^ (Clarivate Analytics). The enrichment identifies statistically significant pathway maps and gene ontology processes associated with this list of genes after false discovery rate (FDR) correction, to summarize the most enriched and pertinent biology associated with the set of genes under investigation (Huang et al., 2009). We also performed enrichment analysis to identify genes differentially expressed at different developmental phases, *via* functional mapping of genetic and expression using the FUMA tool (Watanabe et al., 2017).

#### Cognitive Development Trajectories

With the aim of exploring cognitive development longitudinally, we first run item analysis across different age-based forms of the Bayley Mental Scale using 1-parameter (Rasch) Item Response Theory (IRT). IRT modeling assumes that the probability of a correct response to an item is based only on the ability of the subject and the difficulty of items (Rasch, 1960; de Ayala, 2009), and thereby yields both sample and test independent estimates of item parameters and individual abilities on the latent trait being measured (DeMars, 2010). To scale infant performance for growth interpretations, concurrent vertical scaling was performed taking advantage of an overlapping common item structure (Kolen and Brennan, 2014). This analytical approach provides information on the developmental ordering of items, and the measurement precision associated with the reliability of items and the scores of participants. The calculated separation index shows the scale scores’ capacity to discriminate among children with high, medium, and low ability. The higher the value, the better the separation that exists between the items and between persons and the more precise the representation of the measured ability. Reliability values above 0.80 are considered adequate and separation index above 3 suggests that the scores are sensitive enough to discriminate participants (Linacre, 2010). At this stage cognitive development was estimated using Winsteps Version 3.7 (Linacre, 2010); psychometric properties of the Bayley Mental scaled items and estimates of items’ fit can be found in the Supplementary materials.

Modeling of the cognitive development curve was performed using Linear Mixed Effects Model (LME) (Gałecki and Burzykowski, 2013; Fox and Weisberg, 2019). Models were fitted including the fixed effect of prenatal adversity score, BDNF ePRS, three population stratification principal components, children’s sex, and age at data collection time point, and a quadratic term to model the observed non-linear pattern between age and the outcome. We also considered an interaction term between prenatal adversity, BDNF ePRS, and age. For random effects, participants’ age and the quadratic age term were specified as nested effects with an autoregressive error correlation structure (Fox and Weisberg, 2019), to model individual cognitive development. The pseudo R^2^ for generalized mixed-effect models (Nakagawa and Schielzeth, 2013) was used to compute Cohen’s *f*^2^ measure of local effect size, in which values bellow 0.02 indicate small effect sizes, medium values from 0.02 to 0.15, and values greater than 0.15 are considered large effect (Selya et al., 2012). Packages *lme4* (Pinheiro et al., 2018) and *reghelper* (Hughes, 2020) from R software (R Core Team, 2019) were used to perform the statistical analysis.

#### Structural Magnetic Resonance Imaging

##### Acquisition and Data Preparation

High-resolution T1-weighted images for the whole brain of 47 children from MAVAN cohort were acquired using a 3T Trio Siemens scanner in Montreal and GE MR750 Discovery 3T Magnetic Resonance Imaging (MRI) scanner in Hamilton. We used the following parameters: Montreal) 1 mm isotropic 3D MPRAGE, sagittal acquisition, 256 × 256 mm grid, TR = 2300 ms, TE = 4 ms, FA = 9degrees; Hamilton) a 3D inversion recovery-prepped, T1-weighted anatomical data set, fSPGR, axial acquisition, TE/TR/flip angle = 3.22/10.308/9, 512 × 512 matrix with 1mm slice thickness and 24cm FOV. Computational Anatomy Toolbox (CAT12) from the Statistical Parametric Mapping software (SPM12) was used to process the T1-weighted images. In the preprocessing step, the images were normalized, registered to Montreal Neurological Institute (MNI) space, and segmented into gray matter (GM) and white matter (WM) by voxel-based morphometry. After a high-dimensional Diffeomorphic Anatomical Registration Through Exponentiated Lie Algebra (DARTEL) normalization, that takes into account the sample specific spatial intensity distribution of structural MRI, a smoothing process was applied using 8mm full width half maximum kernel.

##### Parallel Independent Component Analysis

A multivariate Parallel Independent Component Analysis (p-ICA) was performed to identify the relationship between two different data modalities in a data-driven manner (Khadka et al., 2016). In this case, the components of BDNF ePRS (genotype * GTEx gene expression slope for each SNP) and whole-brain voxel-based gray matter density were used. This analysis estimates the maximally independent components within each data modality separately while also maximizing the association between modalities using entropy terms based on information theory (Liu and Calhoun, 2014; Pearlson et al., 2015). This process results in each identified independent component resultant from the p-ICA, being an additive subcomponent of the overall multi variant signal that also considers the relationship with a second data modality The prenatal adversity score was used to define the groups for comparison (23 children high environmental score, 24 children low environmental score), aggregated with the most significant principal components from population stratification for adjustment (ethnicity). The Fusion ICA Toolbox^5^ within MATLAB^®^ R2019 was used to run the analysis. The number of independent components estimated using minimum description length criteria (Calhoun et al., 2010; Pearlson et al., 2015) was 15 for genetic data and 8 for MRI data. The different resulting ICs are interpretable as brain Talairach coordinates are extracted from the MRI components, indicating brain regions that contribute to the overall independent component. As for the genetic modality, the biological relevance of the functionally related SNPs statistically correlated with brain phenotypes is inferred by subsequent enrichment analysis, using annotation software such as the Metacore, thus providing information for interpretation of the genetic independent components. To identify significant brain regions and SNPs that contributed the most to the ICs, IC weights were converted to z-scores and a threshold at | z| > 2.5 was used. Loading coefficients, which describe the presence of the identified component across subjects (Liu et al., 2012), were extracted for each component, modality, and subject. The mean subject-specific loading coefficients of these components between children from high and low prenatal adversity groups were compared using Student’s t-test.

## Results

### Establishment of the Early Life Brain-Derived Neurotrophic Factor Gene Network

The biologically-informed method for selecting SNPs is designed to capture the genes intricately acting in conjunction with the BDNF gene in the prenatal and early life period, hence describing the gene network of interest acting during a specific sensitive period of development. The final list consisted of 46 genes and can be seen in Table 3.

**TABLE 3:** List of genes co-expressed with the BDNF gene and included in the PFC BDNF ePRS.

Gene Symbol	Ensembl	Description	PFC Co-expression Correlation with the BDNF gene in mice
PGD	ENSG00000142657	Phosphogluconate dehydrogenase	–0.81
CBX5	ENSG00000094916	Chromobox 5	0.7
SET	ENSG00000119335	SET nuclear proto-oncogene	0.65
NUP62	ENSG00000213024	Nucleoporin 62	0.63
PFDN2	ENSG00000143256	Prefoldin subunit 2	0.62
SMARCD1	ENSG00000066117	SWI/SNF related, matrix associated, actin dependent regulator of chromatin, subfamily d, member 1	–0.62
CCT4	ENSG00000115484	Chaperonin containing TCP1 subunit 4	0.61
CCT2	ENSG00000166226	Chaperonin containing TCP1 subunit 2	0.60
GTF2F2	ENSG00000188342	General transcription factor IIF subunit 2	0.59
EIF3E	ENSG00000104408	Eukaryotic translation initiation factor 3 subunit E	0.59
SLC39A6	ENSG00000141424	Solute carrier family 39 member 6	–0.59
SEZ6	ENSG00000063015	Seizure related 6 homolog	–0.58
BTG3	ENSG00000154640	BTG anti-proliferation factor 3	0.57
MYCN	ENSG00000134323	MYCN proto-oncogene, bHLH transcription factor	0.57
ODC1	ENSG00000115758	Ornithine decarboxylase 1	0.57
ANTXR2	ENSG00000163297	ANTXR cell adhesion molecule 2	0.56
BCL10	ENSG00000142867	BCL10 immune signaling adaptor	0.56
CCT3	ENSG00000163468	Chaperonin containing TCP1 subunit 3	0.56
MYL12A	ENSG00000101608	Myosin light chain 12A	0.56
SERBP1	ENSG00000142864	SERPINE1 mRNA binding protein 1	0.56
NR4A2	ENSG00000153234	Nuclear receptor subfamily 4 group A member 2	0.55
IGSF9	ENSG00000085552	Immunoglobulin superfamily member 9	0.55
PTPRS	ENSG00000105426	Protein tyrosine phosphatase receptor type S	–0.54
PHF5A	ENSG00000100410	PHD finger protein 5A	0.54
RSL1D1	ENSG00000171490	Ribosomal L1 domain containing 1	0.54
ARF4	ENSG00000168374	ADP ribosylation factor 4	0.54
NFIL3	ENSG00000165030	Nuclear factor, interleukin 3 regulated	0.54
SEC61A1	ENSG00000058262	SEC61 translocon subunit alpha 1	0.53
PSMA2	ENSG00000106588	Proteasome 20S subunit alpha 2	0.53
DNAJB5	ENSG00000137094	DnaJ heat shock protein family (Hsp40) member B5	0.53
GDI2	ENSG00000057608	GDP dissociation inhibitor 2	0.53
NFIB	ENSG00000147862	Nuclear factor I B	–0.53
LMO3	ENSG00000048540	LIM domain only 3	–0.53
RPL11	ENSG00000142676	Ribosomal protein L11	–0.52
NA	ENSG00000155130	Myristoylated alanine rich protein kinase C substrate	0.52
DACT1	ENSG00000165617	Disheveled binding antagonist of beta catenin 1	0.52
KDM6B	ENSG00000132510	Lysine demethylase 6B	0.52
NPM1	ENSG00000181163	Nucleophosmin 1	0.51
CDK8	ENSG00000132964	Cyclin dependent kinase 8	–0.51
OBSCN	ENSG00000154358	Obscurin, cytoskeletal calmodulin and titin-interacting RhoGEF	–0.51
ING1	ENSG00000153487	Inhibitor of growth family member 1	0.50
RBM7	ENSG00000076053	RNA binding motif protein 7	0.50
MTHFD2	ENSG00000065911	Methylenetetrahydrofolate dehydrogenase (NADP + dependent) 2, methenyltetrahydrofolate cyclohydrolase	0.50
BAZ1A	ENSG00000198604	Bromodomain adjacent to zinc finger domain 1A	0.50
SEC11A	ENSG00000140612	SEC11 homolog A, signal peptidase complex subunit	0.50
MMP24	ENSG00000125966	Matrix metallopeptidase 24	–0.50

**FIGURE 1:**
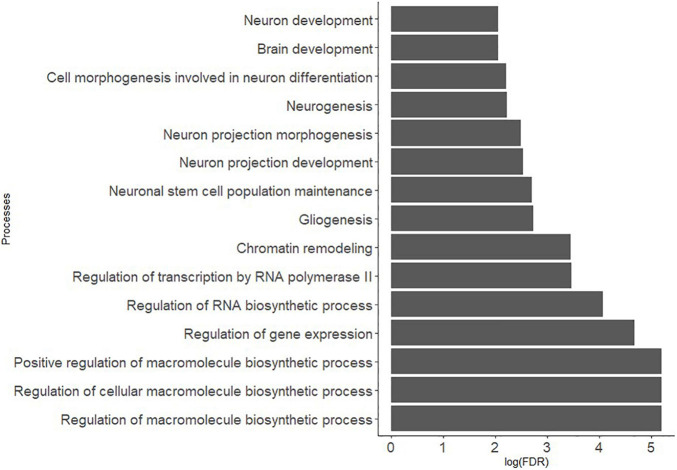
Gene ontology processes related to the genes included in the co-expression PFC BDNF ePRS.

Metacore^®^ enrichment analysis of the 46 genes that contributed to the BDNF ePRS shows false discovery rate (FDR) for pathway maps (Figure 1). Gene ontology processes were enriched for several epigenetic processes, neuron differentiation, and cellular transport. The main biological processes involved in the BDNF ePRS network included biosynthesis of complex macromolecules, regulation of gene expression and RNA transcription, maintenance of neuronal stem cells, neurogenesis, and neuron development.

**FIGURE 2:**
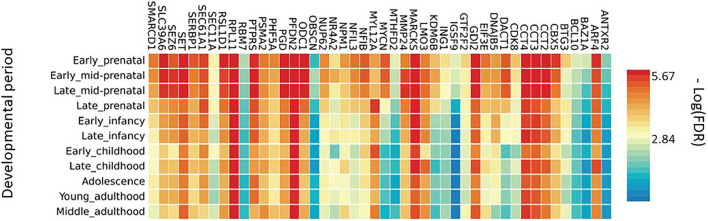
Average gene expression in brain areas at different developmental stages.

To enlighten which genes of the BDNF ePRS are differentially expressed at different developmental phases, we performed a functional mapping of genetic and expression using the FUMA tool (Watanabe et al., 2017). In Figure 2, it is possible to observe that some genes comprising our genetic score have specific expression patterns across distinct developmental periods, suggesting that the function of this gene network varies during development. It is important to notice that our score is enriched for early life developmental periods (transcripts differentially expressed in the PFC in comparison with adult samples), so it is expected that these genes would be highly expressed in early life.

To further understand these different expressions across development, we performed enrichment analysis in the subset of genes that co-variated with age (ARF4, CBX5, CCT2, DACT1, KDM6B, MYCN, MYL12A, NFIB, ODC1, PGD, RSL1D1, SERBP1, SET, SEZ6, SLC39A6, SMARCD1). These genes are significantly enriched for the gene ontology process of regulation of gene expression, DNA transcription, biosynthesis of RNA, and macromolecules. The subset list of genes was also related to chromatin remodeling, axogenesis, and nervous system development.

### Cognitive Development Trajectories

Repeated measures analysis of variance yielded significant mean differences of cognitive development at each time point, *F*(3,468) = 962.8, *P* < 0.001, with significant Bonferroni adjusted p-values for pairwise comparisons between all age groups. Descriptive data on cross-sectional scaled cognitive development at 6, 12, 18, and 36 months are presented in Table 4.

**TABLE 4:** Descriptive statistics of scaled cognitive developmental ability estimates of the Bayley Mental items.

Timepoint	N	Mean	SD
6 months	157	−4.82	13.26
12 months	157	43.61	12.41
18 months	157	100.52	17.72
36 months	157	217.25	16.42

To best characterize the cognitive developmental trajectories from 6 to 36 months we visually inspected the scaled cognitive scores, and data suggested that cognitive skills followed a curvilinear trajectory, which we modeled by adding age quadratic term that reached statistical significance. Our final LME model is presented in Table 5. The model considered growth velocity (age linear term), and acceleration (age quadratic term) of cognitive development, and an interaction effect between BDNF ePRS, prenatal adversity, and age. Neither of the covariates (population stratification components and sex) significantly predicted the outcome.

**TABLE 5:** Results of the linear mixed-effect regression analysis of cognitive developmental trajectories.

	β	SE	*f* ^2^	*P*
Intercept	–12.65	1.66	0.18	<0.001
BDNF ePRS	–3.19	1.35	0.01	0.02
Prenatal Adversity	0.31	0.75	0.03	0.68
BDNF ePRS x Prenatal Adversity	1.73	0.79	0.01	0.03
Age (months)	10.60	0.23	3.20	<0.001
BDNF ePRS x Age	0.17	0.06	0.01	0.01
Prenatal Adversity x Age	–0.14	0.04	0.02	<0.001
BDNF ePRS x Prenatal Adversity x Age	–0.12	0.04	0.02	<0.001
Age quadratic term	–0.07	0.01	0.26	<0.001
Sex female	1.87	1.67	0.00	0.27
PC1	–34.50	20.64	0.01	0.10
PC2	21.31	15.79	0.01	0.18
PC3	–8.11	16.30	0.00	0.62

**FIGURE 3:**
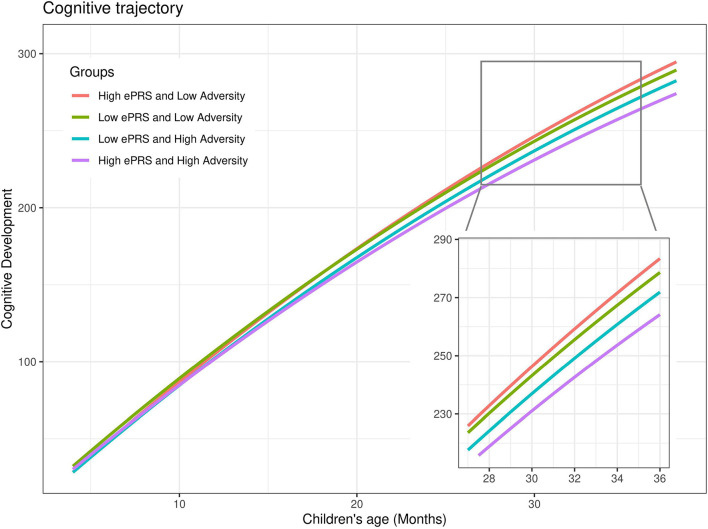
Cognitive developmental growth as function of age, BDNF gene network and cumulative prenatal adversity. Predicted estimates of cognitive development were plotted considering high (+ 1SD) and low (–1SD) BDNF ePRS and high (+ 1SD) and low (–1SD) prenatal adversity for sake of the interaction visualization. Prenatal adversity effects on cognitive development trajectories are larger for children with high BDNF ePRS scores (red vs purple lines) in comparison with children with low BDNF ePRS scores (green and blue lines).

The BDNF ePRS score, prenatal adversity and age presented a significant interaction on cognitive development trajectory (β = −0.12, *P* < 0.001). Cognitive development differences for children with higher BDNF ePRS scores exposed to low and to high prenatal adversity were larger (Figure 3, red line [low adversity] vs purple line [high adversity]) in comparison to children with low BDNF ePRS scores (Figure 3, green line [low adversity] vs blue line [high adversity]). The model shows that, on average, infants with high BDNF genetic scores were more susceptible to prenatal adversity exposure (higher BDNF ePRS and higher prenatal adversity was associated with slower cognitive development trajectory).

### Brain-Derived Neurotrophic Factor Expression Polygenic Score and Gray Matter Associations

Magnetic Resonance Imaging scans from 47 participants at age nine, indicated significant pairs of ICs from two data modalities, the whole-brain voxel-based gray matter density and SNPs from the BDNF ePRS. This means that the pICA identified relationships between the two data modalities, allowing the characterization of the associations between specific portions of our gene network and specific brain regions, suggesting an anatomo-functional basis of the phenotypic differences in neurodevelopmental trajectories. These associations indicated that the genetic IC 10 (G10) was significantly correlated to MRI IC (B6), *r* = −0.65, *p* = 5.76e-07; genetic component 13 (G13) and MRI component (B8), *r* = 0.63, *p* = 1.45e-06; and genetic component 11 (G11) and MRI component 5 (B5), *r* = −0.42, *p* = 2.98e-03.

Comparison of the mean loading coefficients of these three ICs between children from high and low prenatal adversity groups indicated statistically significant differences for G10 (*t* = 2.36, *p* = 0.02), G11 (*t* = −2.05, *p* = 0.04), B8 (*t* = −3.34, *p* = 0.001) and B5 (*t* = 2.09, *p* = 0.04), see Supplementary Table 5. This means that participants from the two prenatal adversity groups contributed differently to the overall IC data pattern.

**FIGURE 4:**
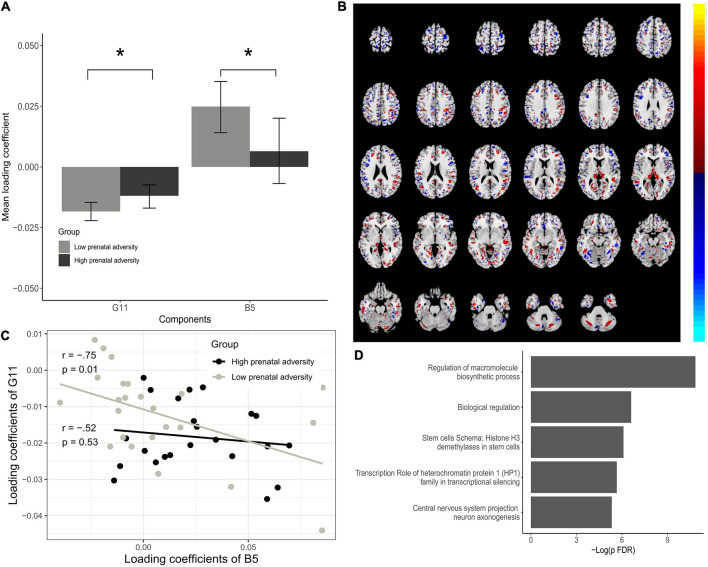
Associations between Gene IC G11 and Brain IC B5. **(A)** Bar plots of estimated loading coefficients for statistically significant brain phenotype (B-5) and genetic component (G11) pair. **(B)** Brain areas comprising the B5 component according to group differences. **(C)** Scatter plot of loading coefficients of B5 and G11 and association between them for low and high prenatal adversity groups. Lines and dots colors represent the High and Low prenatal adversity score groups. **(D)** Enrichment analysis of G11 dominant SNPs. Loading coefficients represent the weight of the overall components for each subject. Significant differences in components are seen contrasting high and low adversity groups, indicating that individuals from high and low adversity groups contributed differently to the overall pattern of the component. *P < 0.05.

The G11-B5 IC pair showed significant differences for both the genetic and brain-phenotype components concerning high and low prenatal adversity and was selected for further analysis. This pair is of primary interest to our study aims and suggests that the relationship between these components is moderated by variations in the quality of the perinatal environment (Figure 4). The G10-B6 and G13-B8 pairs were less informative regarding our main objective, as for the G10-B6 pair only the genetic modality had a significant difference between our groups of interest, and for the G13-B8 pair, only the brain-phenotype component was significant. Brain regions and SNPs comprising these components are described in Supplementary Tables 3, 4.

The interpretation of the significant ICs pair was done by extracting brain Talairach coordinates from MRI IC and by enrichment analysis of the genetic IC, proving interpretable information from the observed patterns. All the significant brain regions for the B5 component as well as for the other brain-phenotype components (B6 and B8) are listed in Supplementary Materials. Many regional variations contribute to the B5 component, located mainly in distinct portions of the occipital, frontal, and parietal cortex. The most prominent regions according to Brodmann areas were 19 and 18 (occipital cortex), 7 (parietal cortex), 6 (frontal cortex) that contributed bilaterally and both negatively and positively to the overall pattern. Brodmann area 10 (anterior PFC) was also associated with the genetic component, although with a less prominent contribution to the overall pattern of the component (Figure 4B). In the G11 component, from the 473 SNPs used, 16 significantly contributed to the component (*Z*-Threshold > ± 2.5). Enrichment analysis showed significant pathway maps related to these SNPs such as the transcription role of heterochromatin protein 1 (HP1) family in transcriptional silencing (FDR = 0.001) and start of DNA replication in early S phase on cell cycle (FDR = 0.018). As for process networks, cell cycle S phase and mitosis (FDR = 0.016) were significant, and gene ontology processes were related to central nervous system development, more specifically commissural neuron axon guidance (FDR = 0.001) and regulation of mRNA processing (FDR = 0.025), response to dsRNA (FDR = 0.011) and negative regulation of transcription by RNA polymerase II (FDR = 0.017). This suggests that variations in gray matter density from the identified regions (from B5) and identified SNPs (from G11) vary together across the sample subjects and that subjects from high and low prenatal adversity groups contribute differently to the overall data pattern of B5 and G11 (Figures 4A,C).

## Discussion

This study aimed at examining the hypothesis that the effects of prenatal exposure to adversity on cognitive trajectories are moderated by the prefrontal BDNF gene network. Differential response to prenatal exposure was captured using a novel bioinformatics approach that provides a biologically-informed genetic score, based on genes co-expressed with the BDNF in the PFC. Significant associations between SNPs weighted by gene expression and gray matter density at 8 to 10 years of age were located mainly in distinct portions of the occipital, frontal, and parietal cortex.

Longitudinally, high BNDF ePRS levels at the PFC were associated with higher environmental susceptibility in predicting the cognitive growth trajectory. Our data support the differential susceptibility model that postulates that individuals that are more likely to be affected by adverse environmental conditions are also most likely to benefit from positive conditions (Belsky, 2013; Belsky et al., 2018). The highest differences were observed in later development (36 months). This result might be related to the delayed messenger RNA expression in the PFC (Somel et al., 2009) and corroborates the enrichment analysis done with FUMA that shows different patterns of gene expression especially at beginning of infancy (Figure 4). Previous research found constancy on BDNF mRNA levels through development in the hippocampus, and variability at the temporal cortex with the highest expression in neonates that decreased with age (Webster et al., 2006).

The neurobiological processes enriched in the BDNF ePRS network were mostly associated with biosynthesis of complex macromolecules, regulation of gene expression and RNA transcription, maintenance of neuronal stem cells, neurogenesis, and neuron development. This is in line with previous animal research that proposes that BDNF system is critically involved in neuron development (Jones et al., 1994), regulation of genes that are associated with synaptic function (Mariga et al., 2015), dendritic growth of cortical neurons (Martin and Finsterwald, 2011), and formation of the neural networks being secreted locally by activity-dependent manner (Hayashi et al., 2007).

Going beyond the analysis of polymorphisms in G × E studies, we integrated information about the gene network of the BDNF with its function at the PFC in a specific developmental period, taking advantage of a cumulative measure of prenatal adversity that reflects a more global level of environmental influence (Silveira et al., 2017; Camerota and Willoughby, 2019). Several mechanisms might be involved in the relation of prenatal adversity and cognitive development, and the observed moderation by the BDNF ePRS. For example, activity-dependent transcription of BDNF is controlled by at least 9 distinct promoters, partially mediated by dynamic changes in DNA methylation (Martinowich et al., 2003; Sakata et al., 2009). Boersma et al. (2013) have found evidence of reduced BDNF expression in response to increased methylation of BDNF at the exon IV in both the amygdala and the hippocampus of prenatally stressed rat’s offspring. In humans, prenatal depressive symptoms were also associated with the BDNF promoter IV region along with NR3C1 1F (Braithwaite et al., 2015), suggesting that this epigenetic marker is developmentally sensitive to the quality of the early environmental exposure (Romens et al., 2015).

Another mechanism that may be involved, is the variability of gene expression in different brain regions at different developmental stages. In order to verify if the genes that composed the PFC BDNF ePRS are co-expressed in infancy and if the list of co-expressed genes is maintained in adulthood, we used databases that included gene expression levels in the human cortex. The heatmap obtained (Figure 2) demonstrates that a high proportion of these genes have different expression levels from early prenatal to late infancy, but others maintained a similar pattern. The pattern of the specific genes that varied from early prenatal to late infancy points to a special role of the network during development, which may be implicated in the relation of early life adversity effect on cognitive development (Gold et al., 2003; Braithwaite et al., 2015).

To further explore the interaction between cumulative prenatal adversity exposure with our genetic scores, we analyzed the association of the BDNF ePRS weighted SNPs in relation and brain matter density considering groups of high x low adversity. The strongest associations were observed at Brodmann areas 19, 6, 18, and 7 that contributed bilaterally and both negatively and positively to the overall pattern. The areas are related to visual association processes, including visual-motor integration, feature extracting, interpretation of images, attentional and multimodal integrating functions, as well as planning of complex and coordinated movements and convergence between vision and proprioception (Gentile et al., 2011; Bear et al., 2016). Significant associations were also found at the anterior PFC (Brodmann area 10). This area is involved in higher-order cognitive functions, for instance, the processing of internal states, strategic processes in memory recall, reallocation of attention, and more broadly the integration of information from across the supramodal cortex (Ramnani and Owen, 2004; Baird et al., 2013), all of which may be associated with the differential responsiveness to environmental adversity, as reflected in our main interaction finding.

Previous research indicates that prenatal exposure to tobacco correlates with a decrease in cortical thickness in the orbitofrontal cortex, in addition to the reduction in BDNF mRNA and protein levels (Lotfipour et al., 2009; Yochum et al., 2014). Experimental and prospective studies have shown that high pregnancy anxiety is negatively associated with gray matter volume, spine density, and dendritic complexity in the PFC (Murmu et al., 2006; Buss et al., 2010) supporting the idea that prenatal adversity has implications at the neurobiological and structural level.

The integration of genotype and gray matter data using p-ICA analysis suggests that environmental conditions have an especial impact on important neurodevelopmental processes. The G11 component is implicated in neural growth, DNA replication, regulation of mRNA processing, and commissural neuron axon guidance. The aforementioned processes are highly susceptible to environmental influences *via* epigenetic factors including DNA methylation and histone acetylation changes (Martinowich et al., 2003; Boulle et al., 2012; Braithwaite et al., 2015). The central role that the BDNF plays in neural development, learning, and memory processes suggests that prenatal exposure to unfavorable intrauterine conditions may compromise proper cognitive function *via* dysfunction of the BDNF system (Jones et al., 1994; Gomez-Pinilla and Vaynman, 2005; Boersma et al., 2013). The disruption of the BDNF network could be even more critical to the more susceptible individuals identified in our study since BDNF function have been repeatedly related to learning and memory, as well as the somatosensory and visual cortices (Gold et al., 2003; Lotfipour et al., 2009; Chiang et al., 2011; Xing et al., 2012). Thus, the observed environmental groups’ differences in common components of gray matter density and the weighted SNPs appear to play a role in a complex phenotype such as cognitive development.

Although long-lasting effects of prenatal adversity exposure were observed in cognitive behavior and gray matter density, we acknowledge that a continued influence of prenatal maternal adversity during the postnatal period is mediated through the quality of mother-infant interactions and the environmental conditions (Monk et al., 2012). The quality of interactions between caregivers and infants during the postnatal period can have a profound impact on several developmental domains predicting neuronal excitability and synaptic plasticity *via* epigenetic pathways (Meaney, 2010; Nguyen et al., 2015; Ohta et al., 2017). It is important to highlight that prenatal negative exposure is not determinant of a negative outcome, but rather offers possible optimistic opportunities for intervention during postnatal development (Bos et al., 2009; Silva et al., 2020).

With this work we expect to contribute with the understanding of how prenatal adversity and the BDNF gene network shape neural and cognitive development, aiming at ultimately inform and improve both prevention and intervention endeavors, yet a few limitations should be addressed. This study would benefit from replication in a different longitudinal cohort specific to the age bands that comprised our sample since during this period children go through several important sensitive periods of development. The smaller sample size of our neuroimaging study is also an aspect that suggests a need for replication using a falsification approach to avoid Type-I errors. Also, PFC subregions have been reported to develop following temporally different trajectories (Shapiro et al., 2017). Therefore, depending on the time when the stressor is applied, distinct effects could be expected in these different subregions, leading to later effects on specific aspects of cognitive behavior. Distinct PFC regions have also been shown to interact differently with the HPA axis: in rodents, GR gene knockdown in the IL cortex potentiated CORT response to a novel stressor in animals previously subjected to chronic stress, while GR knockdown in the PL cortex did not result in the same effect (McKlveen et al., 2013). In addition, functions such as attentional flexibility, reversal learning, and working memory, for example, are dependent on distinct PFC regions (Birrell and Brown, 2000; Manes et al., 2002; McAlonan and Brown, 2003; Gisquet-Verrier and Delatour, 2006). Although exposure to post-natal stress can have opposing effects on dendrite structure and spine density in distinct PFC regions, such as mPFC and OFC (Liston et al., 2006), specific effects of prenatal stress on neuronal structure according to different PFC regions are less studied. Unfortunately, the database (GTEx) used to calculate our polygenic score did not have expression data available from distinct PFC regions. We believe that future studies approaching this point considering specific PFC regions are warranted.

The broader literature on G x E contains few reports of a network approach specific to a determined brain region, use of psychometric modeling to obtain cognitive development trajectories, and the integration of genotype data with neuroimage. We demonstrated that the PFC BDNF gene network moderates the association between exposure to cumulative prenatal adversity and cognitive growth. Our results provide support for the developmental origins of health and disease (DOHaD), along with prenatal fetal programing of biological mechanisms, and differential susceptibility hypotheses (Silveira et al., 2007; Belsky, 2013; Barth et al., 2019). The focus on genes co-expressed with the BDNF allowed us to identify different patterns of enrichment throughout developmental stages that are in line with the multiple sensitive periods of brain development (Knudsen, 2004). It also made it possible to inspect specific pathways more comprehensively than the candidate-gene approach (Silveira et al., 2017). Thus, we expect to contribute to the understanding of neurobiological processes of cognitive development, and how prenatal adversity exerts a long-term influence on this complex phenotype.

## Data Availability Statement

The data are not publicly available due to information that could compromise the privacy of research participants. Requests to access the datasets should be directed to PS.

## Ethics Statement

The studies involving human participants were reviewed and approved by Douglas Hospital Research Centre, Montreal, and St. Joseph Healthcare, Hamilton (protocol number IUSMD-03-45/IUSMD-06-09). Written informed consent to participate in this study was provided by the participants’ legal guardian/next of kin.

## Author Contributions

EM was involved in data analysis, preparation, and review of the manuscript. BB was involved in parallel independent component analysis and preparation of the manuscript. DB was involved in the review of the cognitive development trajectories measure and manuscript. RL, DA, and CD were involved in enrichment analysis and review of the manuscript. IP was involved in preparation, data analysis interpretation, and review of the manuscript. RS and GH were involved in MRI acquisition and processing. MM and PS were involved in the design of study, preparation, and review of the manuscript. All authors contributed to the article and approved the submitted version.

## Conflict of Interest

The authors declare that the research was conducted in the absence of any commercial or financial relationships that could be construed as a potential conflict of interest.

## Publisher’s Note

All claims expressed in this article are solely those of the authors and do not necessarily represent those of their affiliated organizations, or those of the publisher, the editors and the reviewers. Any product that may be evaluated in this article, or claim that may be made by its manufacturer, is not guaranteed or endorsed by the publisher.

## Footnotes

1
http://genenetwork.org

2
http://www.brainspan.org/rnaseq/search/index.html

3
https://www.gtexportal.org/home/

4
https://www.ncbi.nlm.nih.gov/variation/view/

5
http://mialab.mrn.org/software/fit/

## References

[B1] Almas A. N. Degnan K. A. Nelson C. A. Zeanah C. H. Fox N. A. (2016). IQ at Age 12 following a history of Institutional Care: Findings from the Bucharest Early Intervention Project. *Dev. Psychol.* 52 1858–1866. 10.1037/dev0000167 27709994 PMC5083169

[B2] Badihian N. Daniali S. S. Kelishadi R. (2020). Transcriptional and epigenetic changes of brain derived neurotrophic factor following prenatal stress: A systematic review of animal studies. *Neurosci. Biobehav. Rev*. 117 211–231. 10.1016/j.neubiorev.2019.12.018 31838194

[B3] Baird B. Smallwood J. Gorgolewski K. J. Margulies D. S. (2013). Medial and lateral networks in anterior prefrontal cortex support metacognitive ability for memory and perception. *J. Neurosci.* 33 16657–16665. 10.1523/JNEUROSCI.0786-13.2013 24133268 PMC6618531

[B4] Banerjee R. Ghosh A. K. Ghosh B. Bhattacharyya S. Mondal A. C. (2013). Decreased mRNA and protein expression of BDNF, NGF, and their receptors in the hippocampus from suicide: An analysis in human postmortem brain. *Clin. Med. Insights Pathol.* 2013 1–11. 10.4137/CPath.S12530 PMC3767649 24031163

[B5] Barfield E. T. Gourley S. L. (2018). Prefrontal cortical trkB, glucocorticoids, and their interactions in stress and developmental contexts. *Neurosci. Biobehav. Rev*. 95 535–558. 10.1016/j.neubiorev.2018.10.015 30477984 PMC6392187

[B6] Barth B. Portella A. K. Dubé L. Meaney M. J. Silveira P. P. (2019). “The interplay between Dopamine and environment as the biological basis for the early origins of mental health,” in *Early Life Origins of Ageing and Longevity*, ed. Vaiserman A. (New Yor, NY: Springer International Publishing), 121–140. 10.1007/978-3-030-24958-8

[B7] Bartkowska K. Turlejski K. Djavadian R. L. (2010). Neurotrophins and their receptors in early development of the mammalian nervous system. *Acta Neurobiol. Exp.* 2010 454–467. 10.55782/ane-2010-1816 21196952

[B8] Bayley N. (1993). *Bayley Scales of Infant Development: Second Edition*. 2nd ed. San Antonio, TX: The Psychological Corporation.

[B9] Bear M. F. Connors B. W. Paradiso M. A. (2016). *Neuroscience: exploring the brain*. 4th ed. Philadelfia: Wolters Kluwer.

[B10] Belsky J. (2013). Differential Susceptibility to Environmental Influences. *Int. J. Child Care Educ. Policy* 7 15–31. 10.1007/2288-6729-7-2-15

[B11] Belsky J. Pokhvisneva I. Rema A. S. S. Broekman B. F. P. Pluess M. O’Donnell K. J. (2018). Polygenic differential susceptibility to prenatal adversity. *Dev. Psychopathol.* 31 439–441. 10.1017/S0954579418000378 30081968

[B12] Binder D. K. Scharfman H. E. (2004). Brain-derived Neurotrophic Factor. *Growth Factors* 22 123–131. 10.1080/08977190410001723308 15518235 PMC2504526

[B13] Birrell J. M. Brown V. J. (2000). Medial frontal cortex mediates perceptual attentional set shifting in the rat. *J. Neurosci*. 20 4320–4324. 10.1523/jneurosci.20-11-04320.2000 10818167 PMC6772641

[B14] Boersma G. J. Lee R. S. Cordner Z. A. Ewald E. R. Purcell R. H. Moghadam A. A. (2013). Prenatal stress decreases Bdnf expression and increases methylation of Bdnf exon IV in rats. *Epigenetics* 9 437–447. 10.4161/epi.27558 24365909 PMC4053462

[B15] Bos K. J. Fox N. Zeanah C. H. Nelson C. A. (2009). Effects of early psychosocial deprivation on the development of memory and executive function. *Front. Behav. Neurosci.* 3 1–7. 10.3389/neuro.08.016.2009 19750200 PMC2741295

[B16] Boulle F. Van Den Hove D. L. A. Jakob S. B. Rutten B. P. Hamon M. Van Os J. (2012). Epigenetic regulation of the BDNF gene: Implications for psychiatric disorders. *Mol. Psychiatry* 17 584–596. 10.1038/mp.2011.107 21894152

[B17] Bourgeois J.-P. Goldman-Rakic P. S. Rakic P. (1994). Synaptogenesis in the Prefrontal Cortex of Rhesus Monkeys. *Cereb. Cortex* 4 78–96. 10.1093/cercor/4.1.78 8180493

[B18] Braithwaite E. C. Kundakovic M. Ramchandani P. G. Murphy S. E. Champagne F. A. (2015). Maternal prenatal depressive symptoms predict infant NR3C1 1F and BDNF IV DNA methylation. *Epigenetics* 10 408–417. 10.1080/15592294.2015.1039221 25875334 PMC4622733

[B19] Buss C. Davis E. P. Muftuler L. T. Head K. Sandman C. A. (2010). High pregnancy anxiety during mid-gestation is associated with decreased gray matter density in 6-9-year-old children. *Psychoneuroendocrinology* 35 141–153. 10.1016/j.psyneuen.2009.07.010 19674845 PMC2795128

[B20] Calhoun V. D. Adali T. Pearlson G. D. Pekar J. J. (2010). A method for making group inferences from functional MRI data using independent component analysis. *Hum. Brain Mapp.* 933 926–933. 10.1002/hbm 11559959 PMC6871952

[B21] Camerota M. Willoughby M. T. (2019). Prenatal Risk Predicts Preschooler Executive Function: A Cascade Model. *Child Dev.* 00 1–19. 10.1111/cdev.13271 31206640 PMC6917992

[B22] Chen L. M. Yao N. Garg E. Zhu Y. Nguyen T. T. T. Pokhvisneva I. (2018). PRS-on-Spark (PRSoS): a novel, efficient and flexible approach for generating polygenic risk scores. *BMC Bioinform.* 19:295. 10.1186/s12859-018-2289-9 30089455 PMC6083617

[B23] Chiang M. C. Barysheva M. Toga A. W. Medland S. E. Hansell N. K. James M. R. (2011). BDNF gene effects on brain circuitry replicated in 455 twins. *Neuroimage* 55 448–454. 10.1016/j.neuroimage.2010.12.053 21195196 PMC3192852

[B24] Choi B. Leech K. A. Tager-Flusberg H. Nelson C. A. (2018). Development of fine motor skills is associated with expressive language outcomes in infants at high and low risk for autism spectrum disorder. *J. Neurodev. Disord.* 10 1–11. 10.1186/s11689-018-9231-3 29649977 PMC5898056

[B25] Dalle Molle R. Fatemi H. Dagher A. Levitan R. D. Silveira P. P. Dubé L. (2017). Gene and environment interaction: Is the differential susceptibility hypothesis relevant for obesity? *Neurosci. Biobehav. Rev.* 73 326–339. 10.1016/j.neubiorev.2016.12.028 28024828 PMC5283807

[B26] de Ayala R. J. (2009). *The theory and practice of item response theory.* New York, NY: The Guilford Press.

[B27] DeMars C. (2010). *Item response theory. Understanding statistics measurement.* New York, NY: Oxford University Press.

[B28] Drury S. S. Gleason M. M. Theall K. P. Smyke A. T. Nelson C. A. Fox N. A. (2012). Genetic sensitivity to the caregiving context: The influence of 5httlpr and BDNF val66met on indiscriminate social behavior. *Physiol. Behav.* 106 728–735. 10.1016/j.physbeh.2011.11.014 22133521 PMC4084933

[B29] Egan M. F. Kojima M. Callicott J. H. Goldberg T. E. Kolachana B. S. Bertolino A. (2003). The BDNF val66met Polymorphism Affects Activity-Dependent Secretion of BDNF and Human Memory and Hippocampal Function. *Cell* 112 257–269. 10.1016/S0092-8674(03)00035-7 12553913

[B30] Fox J. Weisberg S. (2019). *An R Companion to Applied Regression*, 3nd Edn. Thousand Oaks, CA: SAGE Publications.

[B31] Fuster J. M. (2015). *The prefrontal cortex*, 5th Edn. London: Academic Press.

[B32] Gabrys R. L. Dixon K. Anisman H. (2017). Traumatic Life Events in Relation to Cognitive Flexibility: Moderating Role of the BDNF Val66Met Gene Polymorphism. *Front. Behav. Neurosci*. 11:241. 10.3389/fnbeh.2017.00241 29276480 PMC5727074

[B33] Gaiteri C. Ding Y. French B. Tseng G. C. Sibille E. (2014). Beyond modules and hubs: The potential of gene coexpression networks for investigating molecular mechanisms of complex brain disorders. *Genes Brain Behav.* 13 13–24. 10.1111/gbb.12106 24320616 PMC3896950

[B34] Gałecki A. Burzykowski T. (2013). *Linear Mixed-Effects Models Using R: A Step-by-Step Approach.* New York, NY: Springer.

[B35] Gentile G. Petkova V. I. Ehrsson H. H. (2011). Integration of visual and tactile signals from the hand in the human brain: An fMRI study. *J. Neurophysiol.* 105 910–922. 10.1152/jn.00840.2010 21148091 PMC3059180

[B36] Gisquet-Verrier P. Delatour B. (2006). The role of the rat prelimbic/infralimbic cortex in working memory: not involved in the short-term maintenance but in monitoring and processing functions. *Neuroscience* 141 585–596. 10.1016/j.neuroscience.2006.04.009 16713111

[B37] Gold B. Dean M. Zaitsev E. Kojima M. Egan M. F. Goldman D. (2003). The BDNF val66met Polymorphism Affects Activity-Dependent Secretion of BDNF and Human Memory and Hippocampal Function. *Cell* 112 257–269. 10.1016/s0092-8674(03)00035-7 12553913

[B38] Gomez-Pinilla F. Vaynman S. (2005). A “deficient environment” in prenatal life may compromise systems important for cognitive function by affecting BDNF in the hippocampus. *Exp. Neurol.* 192 235–243. 10.1016/j.expneurol.2004.12.001 15755541

[B39] Gorski J. Balogh S. Wehner J. Jones K. (2003). Learning deficits in forebrain-restricted brain-derived neurotrophic factor mutant mice. *Neuroscience* 121 341–354. 10.1016/S0306-4522(03)00426-3 14521993

[B40] Gorski J. A. Zeiler S. R. Tamowski S. Jones K. R. (2003). Brain-Derived Neurotrophic Factor Is Required for the Maintenance of Cortical Dendrites. *J. Neurosci*. 23 6856–6865. 10.1523/JNEUROSCI.23-17-06856.2003 12890780 PMC6740724

[B41] Gunnar M. R. Wenner J. A. Thomas K. M. Glatt C. E. McKenna M. C. Clark A. G. (2012). The brain-derived neurotrophic factor Val66Met polymorphism moderates early deprivation effects on attention problems. *Dev. Psychopathol.* 24 1215–1223. 10.1017/S095457941200065X 23062292 PMC3581017

[B42] Haeussler M. Kriegstein A. R. Alvarado B. Ounadjela J. R. Bhaduri A. West J. A. (2017). Spatiotemporal gene expression trajectories reveal developmental hierarchies of the human cortex. *Science* 358 1318–1323. 10.1126/science.aap8809 29217575 PMC5991609

[B43] Hair N. L. Hanson J. L. Wolfe B. L. Pollak S. D. (2015). Association of child poverty, brain development, and academic achievement. *JAMA Pediatr.* 169 822–829. 10.1001/jamapediatrics.2015.1475 26192216 PMC4687959

[B44] Hari Dass S. A. McCracken K. Pokhvisneva I. Chen L. M. Garg E. Nguyen T. T. T. (2019). A biologically-informed polygenic score identifies endophenotypes and clinical conditions associated with the insulin receptor function on specific brain regions. *EBioMedicine* 2019:51. 10.1016/j.ebiom.2019.03.051 30922963 PMC6491717

[B45] Hayashi A. Kasahara T. Iwamoto K. Ishiwata M. Kametani M. Kakiuchi C. (2007). The role of Brain-derived Neurotrophic Factor (BDNF)-induced XBP1 splicing during brain development. *J. Biol. Chem.* 282 34525–34534. 10.1074/jbc.M704300200 17890727

[B46] Hemmerle A. M. Ahlbrand R. Bronson S. L. Lundgren K. H. Richtand N. M. Seroogy K. B. (2015). Modulation of schizophrenia-related genes in the forebrain of adolescent and adult rats exposed to maternal immune activation. *Schizophr. Res*. 168 411–420. 10.1016/j.schres.2015.07.006 26206493 PMC4591187

[B47] Hempstead B. L. (2014). “Deciphering proneurotrophin actions,” in *Neurotrophic Factors*, eds Lewin G. R. Carter B. D. (Berlin: Springer), 17–32. 10.1007/978-3-642-45106-5 24668468

[B48] Hoops D. Flores C. (2017). Making Dopamine Connections in Adolescence. *Trends Neurosci*. 40 709–719. 10.1016/j.tins.2017.09.004 29032842 PMC5705341

[B49] Huang D. W. Sherman B. T. Lempicki R. A. (2009). Bioinformatics enrichment tools: Paths toward the comprehensive functional analysis of large gene lists. *Nucleic Acids Res.* 37 1–13. 10.1093/nar/gkn923 19033363 PMC2615629

[B50] Hughes J. (2020). *reghelper: Helper Functions for Regression Analysis.* Available online at: https://cran.r-project.org/package=reghelper (accessed date 2021-02-20).

[B51] Ji Y. Pang P. T. Feng L. Lu B. (2005). Cyclic AMP controls BDNF-induced TrkB phosphorylation and dendritic spine formation in mature hippocampal neurons. *Nat. Neurosci*. 8 164–172. 10.1038/nn1381 15665879

[B52] Jones K. R. Fariñas I. Backus C. Reichardt L. F. (1994). Targeted disruption of the BDNF gene perturbs brain and sensory neuron development but not motor neuron development. *Cell* 76 989–999. 10.1016/0092-8674(94)90377-8 8137432 PMC2711896

[B53] Khadka S. Pearlson G. D. Calhoun V. D. Liu J. Gelernter J. Bessette K. L. (2016). Multivariate imaging genetics study of MRI gray matter volume and SNPs reveals biological pathways correlated with brain structural differences in attention deficit hyperactivity disorder. *Front. Psychiatry* 7:128. 10.3389/fpsyt.2016.00128 27504100 PMC4959119

[B54] Knudsen E. (2004). Sensitive periods in the development of the brain and behavior. *J. Cogn. Neurosci.* 2004 1412–1425. 10.1162/0898929042304796 15509387

[B55] Kolb B. Mychasiuk R. Muhammad A. Li Y. Frost D. O. Gibb R. (2012). Experience and the developing prefrontal cortex. *Proc. Natl. Acad. Sci.* 109 17186–17193. 10.1073/pnas.1121251109 23045653 PMC3477383

[B56] Kolen M. J. Brennan R. L. (2014). *Test Equating, Scaling, and Linking: Methods and Practices*. 3rd. New York, NY: Springer.

[B57] Kowiański P. Lietzau G. Czuba E. Waśkow M. Steliga A. Moryś J. (2018). BDNF: A Key Factor with Multipotent Impact on Brain Signaling and Synaptic Plasticity. *Cell. Mol. Neurobiol.* 38 579–593. 10.1007/s10571-017-0510-4 28623429 PMC5835061

[B58] Linacre J. M. (2010). *A User’s Guide to Winsteps: Rasch-Model Computer Programs.* Available online at: www.winsteps.com (accessed date February 14, 2021).

[B59] Liston C. Miller M. M. Goldwater D. S. Radley J. J. Rocher A. B. Hof P. R. (2006). Stress-induced alterations in prefrontal cortical dendritic morphology predict selective impairments in perceptual attentional set-shifting. *J. Neurosci*. 26 7870–7874. 10.1523/JNEUROSCI.1184-06.2006 16870732 PMC6674229

[B60] Liu J. Calhoun V. D. (2014). A review of multivariate analyses in imaging genetics. *Front. Neuroinform.* 8 1–11. 10.3389/fninf.2014.00029 24723883 PMC3972473

[B61] Liu J. Ghassemi M. M. Michael A. M. Boutte D. Wells W. Perrone-Bizzozero N. (2012). An ICA with reference approach in identification of genetic variation and associated brain networks. *Front. Hum. Neurosci.* 6 1–10. 10.3389/fnhum.2012.00021 22371699 PMC3284145

[B62] Lobel M. Dunkel-schetter C. (1990). Conceptualizing stress to study effects on health: Environmental, perceptual, and emotional components. *Anxiety Res.* 3 213–230. 10.1080/08917779008248754

[B63] Lotfipour S. Ferguson E. Leonard G. Perron M. Pike B. Richer L. (2009). Orbitofrontal Cortex and Drug Use During Adolescence: Role of prenatal exposure to maternal smoking and BDNF genotype. *Arch. Gen. Psychiatry* 66:1244. 10.1001/archgenpsychiatry.2009.124 19884612

[B64] Lu H. Park H. Poo M.-M. (2014). Spike-timing-dependent BDNF secretion and synaptic plasticity. *Philos. Trans. R. Soc. B Biol. Sci*. 369:20130132. 10.1098/rstb.2013.0132 24298135 PMC3843865

[B65] Manes F. Sahakian B. Clark L. Rogers R. Antoun N. Aitken M. (2002). Decision-making processes following damage to the prefrontal cortex. *Brain* 125 624–639. 10.1093/brain/awf049 11872618

[B66] Mariga A. Zavadil J. Ginsberg S. D. Chao M. V. (2015). Withdrawal of BDNF from hippocampal cultures leads to changes in genes involved in synaptic function. *Dev. Neurobiol.* 75 173–192. 10.1002/dneu.22216 25059794 PMC4329925

[B67] Martin J. L. Finsterwald C. (2011). Cooperation between BDNF and glutamate in the regulation of synaptic transmission and neuronal development. *Commun. Integr. Biol.* 4 1–3. 10.4161/cib.4.1.13761 21509169 PMC3073261

[B68] Martinowich K. Hattori D. Wu H. Fouse S. He F. Hu Y. (2003). DNA methylation-related chromatin remodeling in activity-dependent BDNF gene regulation. *Science* 302 890–893. 10.1126/science.1090842 14593184

[B69] Matrisciano F. Tueting P. Maccari S. Nicoletti F. Guidotti A. (2012). Pharmacological Activation of Group-II Metabotropic Glutamate Receptors Corrects a Schizophrenia-Like Phenotype Induced by Prenatal Stress in Mice. *Neuropsychopharmacology* 37 929–938. 10.1038/npp.2011.274 22089319 PMC3280642

[B70] McAlonan K. Brown V. J. (2003). Orbital prefrontal cortex mediates reversal learning and not attentional set shifting in the rat. *Behav. Brain Res*. 146 97–103. 10.1016/j.bbr.2003.09.019 14643463

[B71] McCarthy S. Das S. Kretzschmar W. Delaneau O. Wood A. R. Teumer A. (2016). A reference panel of 64,976 haplotypes for genotype imputation. *Nat. Genet.* 48 1279–1283. 10.1038/ng.3643 27548312 PMC5388176

[B72] McKlveen J. M. Myers B. Flak J. N. Bundzikova J. Solomon M. B. Seroogy K. B. (2013). Role of Prefrontal Cortex Glucocorticoid Receptors in Stress and Emotion. *Biol. Psychiatry* 74 672–679. 10.1016/j.biopsych.2013.03.024 23683655 PMC3797253

[B73] Meaney M. J. (2010). Epigenetics and the biological definition of gene X environment interactions. *Child Dev.* 81 41–79. 10.1111/j.1467-8624.2009.01381.x 20331654

[B74] Molendijk M. L. Bus B. A. Spinhoven P. Kaimatzoglou A. Voshaar R. C. O. Penninx B. W. (2012). A systematic review and meta-analysis on the association between BDNF val66met and hippocampal volume-A genuine effect or a winners curse? *Am. J. Med. Genet. Part B Neuropsychiatr. Genet.* 159 731–740. 10.1002/ajmg.b.32078 22815222

[B75] Monk C. Spicer J. Champagne F. A. (2012). Linking Prenatal Maternal Adversity to Developmental Outcomes in Infants: The Role of Epigenetic Pathways. *Dev. Psychopathol.* 24 1361–1376. 10.1017/S0954579412000764.Linking 23062303 PMC3730125

[B76] Murmu M. S. Salomon S. Biala Y. Weinstock M. Braun K. Bock J. (2006). Changes of spine density and dendritic complexity in the prefrontal cortex in offspring of mothers exposed to stress during pregnancy. *Eur. J. Neurosci.* 24 1477–1487. 10.1111/j.1460-9568.2006.05024.x 16965544

[B77] Nagata T. Shinagawa S. Nukariya K. Yamada H. Nakayama K. (2012). Association between BDNF Polymorphism (Val66Met) and Executive Function in Patients with Amnestic Mild Cognitive Impairment or Mild Alzheimer Disease. *Dement. Geriatr. Cogn. Disord*. 33 266–272. 10.1159/000339358 22699449

[B78] Nakagawa S. Schielzeth H. (2013). A general and simple method for obtaining R2 from generalized linear mixed-effects models. *Methods Ecol. Evol.* 4 133–142. 10.1111/j.2041-210x.2012.00261.x

[B79] Nelson C. A. Gabard-Durnam L. J. (2020). Early Adversity and Critical Periods: Neurodevelopmental Consequences of Violating the Expectable Environment. *Trends Neurosci.* 43 133–143. 10.1016/j.tins.2020.01.002 32101708 PMC8092448

[B80] Newberger E. H. Barkan S. E. Lieberman E. S. McCormick M. C. Yllo K. Gary L. T. (1992). Abuse of pregnant women and adverse birth outcome: Current knowledge and implications for practice. *J. ofthe Am. Med. Assoc.* 267 2370–2372. 10.1001/jama.1992.03480170096037 1564780

[B81] Nguyen H. B. Bagot R. C. Diorio J. Wong T. P. Meaney M. J. (2015). Maternal care differentially affects neuronal excitability and synaptic plasticity in the dorsal and ventral hippocampus. *Neuropsychopharmacology* 40 1590–1599. 10.1038/npp.2015.19 25598429 PMC4915255

[B82] Niu Y. Wang T. Liang S. Li W. Hu X. Wu X. (2020). Sex−dependent aberrant PFC development in the adolescent offspring rats exposed to variable prenatal stress. *Int. J. Dev. Neurosci*. 80 464–476. 10.1002/jdn.10034 32358823

[B83] Noble K. G. Houston S. M. Brito N. H. Bartsch H. Kan E. Kuperman J. M. (2015). Family income, parental education and brain structure in children and adolescents. *Nat. Neurosci.* 18 773–778. 10.1038/nn.3983 25821911 PMC4414816

[B84] Numakawa T. Kumamaru E. Adachi N. Yagasaki Y. Izumi A. Kunugi H. (2009). Glucocorticoid receptor interaction with TrkB promotes BDNF-triggered PLC- signaling for glutamate release *via* a glutamate transporter. *Proc. Natl. Acad. Sci*. 106 647–652. 10.1073/pnas.0800888106 19126684 PMC2626757

[B85] O’Donnell K. A. Gaudreau H. Colalillo S. Steiner M. Atkinson L. Moss E. (2014). The maternal adversity, vulnerability and neurodevelopment project: Theory and methodology. *Can. J. Psychiatry* 59 497–508. 10.1177/070674371405900906 25565695 PMC4168812

[B86] O’Donnell K. J. Glover V. Barker E. D. O’Connor T. G. (2014a). The persisting effect of maternal mood in pregnancy on childhood psychopathology. *Dev. Psychopathol.* 26 393–403. 10.1017/s0954579414000029 24621564

[B87] O’Donnell K. J. Glover V. Holbrook J. D. O’Connor T. G. (2014b). Maternal prenatal anxiety and child brain-derived neurotrophic factor (BDNF) genotype: Effects on internalizing symptoms from 4 to 15 years of age. *Dev. Psychopathol.* 26 1255–1266. 10.1017/S095457941400100X 25422959

[B88] Ohta K. I. Suzuki S. Warita K. Kaji T. Kusaka T. Miki T. (2017). Prolonged maternal separation attenuates BDNF-ERK signaling correlated with spine formation in the hippocampus during early brain development. *J. Neurochem.* 141 179–194. 10.1111/jnc.13977 28178750

[B89] Pearlin L. I. Schooler C. (1978). The structure of coping. *J. Health Soc. Behav.* 19 2–21. 10.1016/j.jns.2003.09.014 649936

[B90] Pearlson G. D. Liu J. Calhoun V. D. (2015). An introductory review of parallel independent component analysis (p-ICA) and a guide to applying p-ICA to genetic data and imaging phenotypes to identify disease-associated biological pathways and systems in common complex disorders. *Front. Genet.* 6 1–13. 10.3389/fgene.2015.00276 26442095 PMC4561364

[B91] Pinheiro J. Bates D. DebRoy S. Sarkar D. (2018). *nlme: Linear and Nonlinear Mixed Effects Models.* Available online at: https://cran.r-project.org/package=nlme (accessed date 2021-09-07).

[B92] Price A. L. Patterson N. J. Plenge R. M. Weinblatt M. E. Shadick N. A. Reich D. (2006). Principal components analysis corrects for stratification in genome-wide association studies. *Nat. Genet.* 38 904–909. 10.1038/ng1847 16862161

[B93] Purcell S. Neale B. Todd-Brown K. Thomas L. Ferreira M. A. R. Bender D. (2007). PLINK: A Tool Set for Whole-Genome Association and Population-Based Linkage Analyses. *Am. J. Hum. Genet*. 81 559–575. 10.1086/519795 17701901 PMC1950838

[B94] R Core Team (2019). *R: A language and environment for statistical computing.* Vienna: R Core Team.

[B95] Radolf L. S. (1977). The CES-D Scale A Self report Depression Scale for Research in the General Population. *Appl. Psychol. Meas.* 1 385–401. 10.1177/014662167700100306 26918431

[B96] Ramnani N. Owen A. M. (2004). Anterior prefrontal cortex: Insights into function from anatomy and neuroimaging. *Nat. Rev. Neurosci.* 5 184–194. 10.1038/nrn1343 14976518

[B97] Rasch G. (1960). *Probabilistic models for some intelligence and attainement tests.* Copenhagen: Danmarks Paedogogiske Institut.

[B98] Reinhart V. Bove S. E. Volfson D. Lewis D. A. Kleiman R. J. Lanz T. A. (2015). Evaluation of TrkB and BDNF transcripts in prefrontal cortex, hippocampus, and striatum from subjects with schizophrenia, bipolar disorder, and major depressive disorder. *Neurobiol. Dis.* 77 220–227. 10.1016/j.nbd.2015.03.011 25796564

[B99] Romens S. E. Mcdonald J. Svaren J. Pollak S. D. (2015). Associations Between Early Life Stress and Gene Methylation in Children. *Child Dev.* 86 303–309. 10.1111/cdev.12270 25056599 PMC4305348

[B100] Roth T. L. Lubin F. D. Funk A. J. Sweatt J. D. (2009). Lasting Epigenetic Influence of Early-Life Adversity on the BDNF Gene. *Biol. Psychiatry* 65 760–769. 10.1016/j.biopsych.2008.11.028 19150054 PMC3056389

[B101] Roth T. L. Zoladz P. R. Sweatt J. D. Diamond D. M. (2011). Epigenetic modification of hippocampal Bdnf DNA in adult rats in an animal model of post-traumatic stress disorder. *J. Psychiatr. Res.* 45 919–926. 10.1016/j.jpsychires.2011.01.013 21306736 PMC3335738

[B102] Sakata K. Woo N. H. Martinowich K. Greene J. S. Schloesser R. J. Shen L. (2009). Critical role of promoter IV-driven BDNF transcription in GABAergic transmission and synaptic plasticity in the prefrontal cortex. *Proc. Natl. Acad. Sci.* 106 5942–5947. 10.1073/pnas.0811431106 19293383 PMC2667049

[B103] Selya A. S. Rose J. S. Dierker L. C. Hedeker D. Mermelstein R. J. (2012). A practical guide to calculating Cohen’s f 2, a measure of local effect size, from PROC MIXED. *Front. Psychol.* 3 1–6. 10.3389/fpsyg.2012.00111 22529829 PMC3328081

[B104] Shapiro L. P. Parsons R. G. Koleske A. J. Gourley S. L. (2017). Differential expression of cytoskeletal regulatory factors in the adolescent prefrontal cortex: Implications for cortical development. *J. Neurosci. Res*. 95 1123–1143. 10.1002/jnr.23960 27735056 PMC5352542

[B105] Shaw G. A. Dupree J. L. Neigh G. N. (2020). Adolescent maturation of the prefrontal cortex: Role of stress and sex in shaping adult risk for compromise. *Genes, Brain Behav*. 19:12626. 10.1111/gbb.12626 31769158

[B106] Silva M. A. da, de Mendonça Filho E. J. Mônego B. G. Bandeira D. R. (2020). Instruments for multidimensional assessment of child development: a systematic review. *Early Child Dev. Care* 190 1257–1271. 10.1080/03004430.2018.1528243

[B107] Silveira P. P. Pokhvisneva I. Parent C. Cai S. Rema A. S. S. Broekman B. F. P. (2017). Cumulative prenatal exposure to adversity reveals associations with a broad range of neurodevelopmental outcomes that are moderated by a novel, biologically informed polygenetic score based on the serotonin transporter solute carrier family C6, member 4. *Dev. Psychopathol.* 29 1601–1617. 10.1017/s0954579417001262 29162172

[B108] Silveira P. P. Portella A. K. Goldani M. Z. Barbieri M. A. (2007). Developmental origins of health and disease (DOHaD). *J. Pediatr.* 83 494–504. 10.2223/JPED.1728 18074050

[B109] Sobolewski M. Abston K. Conrad K. Marvin E. Harvey K. Susiarjo M. (2020). Lineage-and sex-dependent behavioral and biochemical transgenerational consequences of developmental exposure to lead, prenatal stress, and combined lead and prenatal stress in mice. *Environ. Health Perspect*. 128 1–14. 10.1289/EHP4977 32073883 PMC7064322

[B110] Somel M. Franz H. Yan Z. Lorenc A. Guo S. Giger T. (2009). Transcriptional neoteny in the human brain. *Proc. Natl. Acad. Sci.* 106 5743–5748. 10.1073/pnas.0900544106 19307592 PMC2659716

[B111] Spielberger C. D. (1989). *Manual for the State-Trait Anxiety Inventory.* Palo Alto, CA: Consulting Psychologists Press.

[B112] The GTEx Consortium (2013). The Genotype-Tissue Expression (GTEx) project. *Nat. Genet.* 45 580–585. 10.1038/ng.2653 23715323 PMC4010069

[B113] Tottenham N. (2019). Early Adversity and the Neotenous Human Brain. *Biol. Psychiatry* 2019 1–10. 10.1016/j.biopsych.2019.06.018 31399257 PMC6935437

[B114] Watanabe K. Taskesen E. Van Bochoven A. Posthuma D. (2017). Functional mapping and annotation of genetic associations with FUMA. *Nat. Commun.* 8 1–10. 10.1038/s41467-017-01261-5 29184056 PMC5705698

[B115] Webster M. J. Herman M. M. Kleinman J. E. Shannon Weickert C. (2006). BDNF and trkB mRNA expression in the hippocampus and temporal cortex during the human lifespan. *Gene Expr. Patterns* 6 941–951. 10.1016/j.modgep.2006.03.009 16713371

[B116] West A. E. Pruunsild P. Timmusk T. (2014). “Neurotrophins: Transcription and translation,” in *Neurotrophic Factors*, eds Lewin G. R. Carter B. D. (Berlin: Springer), 67–100. 10.1007/978-3-642-45106-5_4 24668470

[B117] Xing B. Guo J. Meng X. Wei S. G. Li S. Bin (2012). The dopamine D1 but not D3 receptor plays a fundamental role in spatial working memory and BDNF expression in prefrontal cortex of mice. *Behav. Brain Res.* 235 36–41. 10.1016/j.bbr.2012.06.035 22776159

[B118] Xu B. Zang K. Ruff N. L. Zhang Y. A. McConnell S. K. Stryker M. P. (2000). Cortical Degeneration in the Absence of Neurotrophin Signaling. *Neuron* 26 233–245. 10.1016/S0896-6273(00)81153-8 10798407

[B119] Yochum C. Doherty-Lyon S. Hoffman C. Hossain M. M. Zelikoff J. T. Richardson J. R. (2014). Prenatal cigarette smoke exposure causes hyperactivity and aggressive behavior: Role of altered catecholamines and BDNF. *Exp. Neurol.* 254 145–152. 10.1016/j.expneurol.2014.01.016 24486851 PMC3982151

[B120] Zhang Z. Fan J. Ren Y. Zhou W. Yin G. (2013). The release of glutamate from cortical neurons regulated by BDNF *via* the TrkB/Src/PLC-γ1 pathway. *J. Cell. Biochem.* 114 144–151. 10.1002/jcb.24311 22886995

