# Supplementary material for: Cognitive Development and Brain Gray Matter Susceptibility to Prenatal Adversities: Moderation by the Prefrontal Cortex Brain-Derived Neurotrophic Factor Gene Co-expression Network
Source: Front Neurosci. 2021 Nov 24;15:744743. doi: 10.3389/fnins.2021.744743 (PMC8652300; doi:10.3389/fnins.2021.744743)
Supplement: Supplementary file 1 [file Presentation_1.pdf]

Supplementary Materials for

Cognitive development and brain gray matter susceptibility to prenatal adversities: moderation by the prefrontal cortex BDNF gene co-expression network

Euclides José de Mendonça Filho, Barbara Barth, Denise Ruschel Bandeira, Randriely Merscher Sobreira de Lima, Danusa Mar Arcego, Carla Dalmaz, Irina Pokhvisneva, Roberto Britto Sassi, Geoffrey B. C. Hall, Michael J. Meaney, Patricia Pelufo Silveira*

***Correspondence:** patricia.silveira@mcgill.ca

This document includes:

Supplementary Materials and Methods

Supplementary Results

Figure S1

Table S1 to S5

SUPPLEMENTARY MATERIALS AND METHODS

Psychometric properties of the cognitive development measure

Item response theory scaled items from the Bayley Mental scale had a separation index of 19.3 (Rasch reliability of 1.00), indicating that the sample of young children could be scaled into a comparable cognitive continuum. Overall infit mean-square for items was .97. Results suggest that items provided a high degree of precision for detecting small differences in ability among children (see Table S1 for psychometric properties). The item-person map shows the developmental pathway of items; it is possible to observe that items were more informative of abilities of the age-appropriate groups (Figure S1). This analysis confirms that in our sample Bayley mental items are adequately measuring cognitive development.

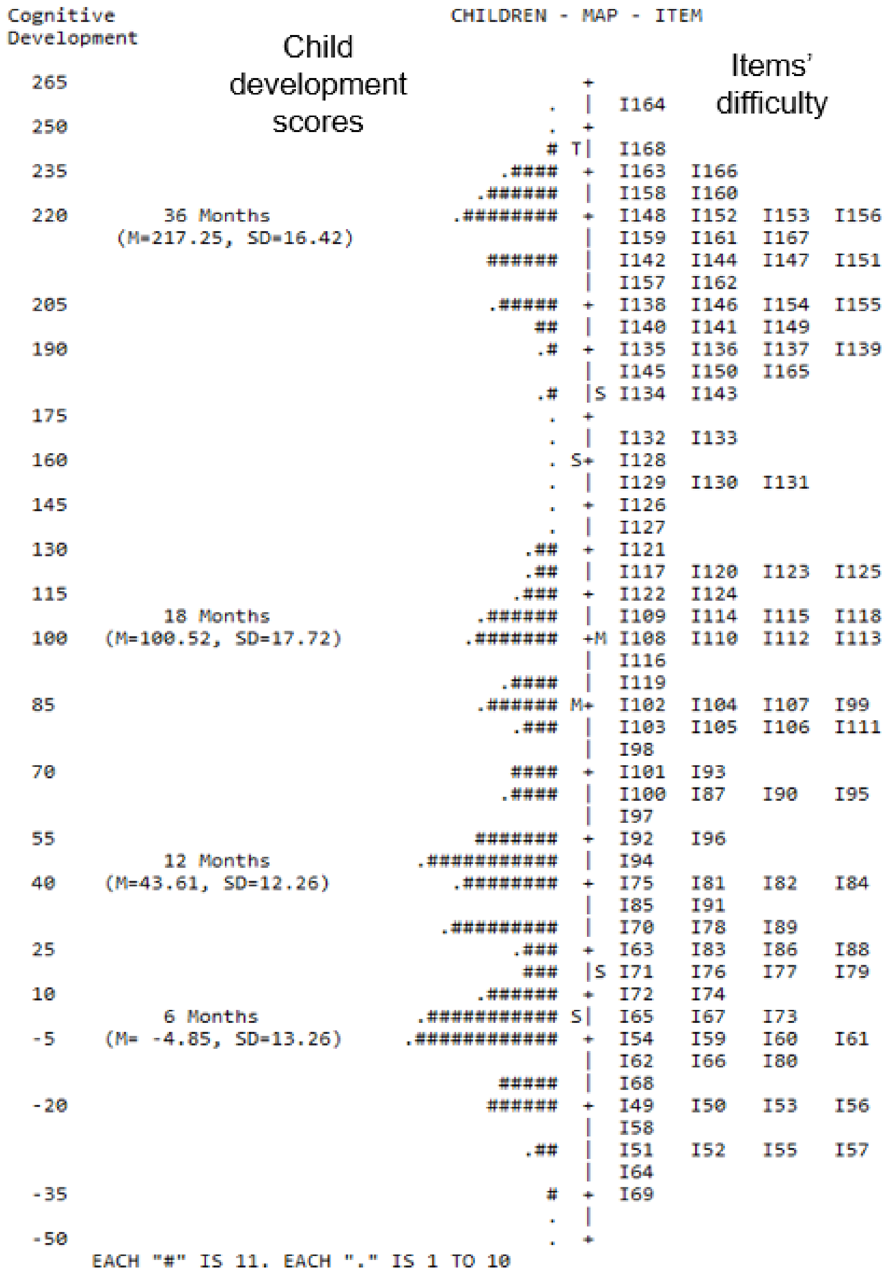

Figure S1. Person-item map of the Bayley Mental scale.

Table S1. *Rasch analysis parameters of the Bayley Mental scale items (BSID-II)*

Item	θ	Infit	Correlation	Error	Item	θ	Infit	Correlation	Error	Item	θ	Infit	Correlation	Error	Item	θ	Infit	Correlation	Error
Q49	-8.18	0.92	0.44	0.39	Q79	-5.66	1.05	0.25	0.35	Q109	0.51	0.89	0.59	0.29	Q139	6.06	0.69	0.19	0.05
Q50	-8.01	0.89	0.47	0.40	Q80	-6.78	1.01	0.18	0.23	Q110	0.18	0.89	0.63	0.29	Q140	6.34	0.78	0.76	0.16
Q51	-8.40	1.14	0.17	0.41	Q81	-4.24	1.00	0.36	0.45	Q111	-1.39	0.96	0.80	0.27	Q141	6.31	0.97	0.76	0.18
Q52	-8.71	1.09	0.22	0.38	Q82	-4.10	1.03	0.33	0.46	Q112	-0.12	1.06	0.65	0.32	Q142	7.38	1.35	0.63	0.24
Q53	-8.11	0.95	0.41	0.40	Q83	-4.84	1.11	0.21	0.44	Q113	-0.13	0.78	0.69	0.28	Q143	5.73	0.88	0.80	0.15
Q54	-6.79	0.92	0.46	0.44	Q84	-4.01	1.06	0.31	0.47	Q114	0.65	0.96	0.56	0.29	Q144	7.31	0.92	0.66	0.20
Q55	-8.66	0.98	0.34	0.36	Q85	-3.95	1.08	0.27	0.48	Q115	0.48	1.01	0.57	0.31	Q145	5.87	0.83	0.79	0.15
Q56	-8.01	1.09	0.25	0.44	Q86	-4.79	0.89	0.46	0.40	Q116	-0.08	1.17	0.63	0.34	Q146	7.05	0.89	0.70	0.19
Q57	-8.74	1.05	0.23	0.37	Q87	-2.69	0.91	0.45	0.40	Q117	1.26	0.93	0.49	0.27	Q147	7.62	1.14	0.62	0.22
Q58	-7.89	0.92	0.45	0.41	Q88	-4.78	1.05	0.28	0.43	Q118	0.48	0.97	0.58	0.30	Q148	7.81	1.02	0.60	0.21
Q59	-6.85	0.92	0.46	0.44	Q89	-4.26	0.94	0.42	0.44	Q119	-0.42	1.07	0.68	0.32	Q149	6.75	1.05	0.72	0.20
Q60	-6.77	1.00	0.37	0.46	Q90	-2.70	1.01	0.33	0.43	Q120	1.48	1.02	0.44	0.27	Q150	5.90	1.02	0.79	0.17
Q61	-7.09	0.94	0.45	0.45	Q91	-3.99	0.96	0.42	0.45	Q121	2.04	0.86	0.40	0.22	Q151	7.51	0.94	0.64	0.20
Q62	-7.08	0.93	0.45	0.45	Q92	-2.99	1.03	0.33	0.45	Q122	1.12	0.84	0.52	0.26	Q152	8.01	0.94	0.58	0.20
Q63	-5.10	1.02	0.25	0.36	Q93	-1.98	1.05	0.23	0.37	Q123	1.27	1.16	0.45	0.30	Q153	8.03	0.83	0.58	0.19
Q64	-8.41	0.93	0.42	0.37	Q94	-3.45	1.02	0.35	0.46	Q124	0.93	0.97	0.52	0.29	Q154	6.89	0.87	0.71	0.19
Q65	-6.65	0.91	0.47	0.44	Q95	-2.46	0.94	0.39	0.39	Q125	1.71	0.87	0.44	0.24	Q155	6.97	0.95	0.70	0.20
Q66	-7.14	0.94	0.44	0.45	Q96	-2.97	0.98	0.37	0.44	Q126	3.04	0.86	0.29	0.16	Q156	8.23	0.94	0.55	0.20
Q67	-6.28	1.04	0.31	0.46	Q97	-2.44	1.00	0.66	0.36	Q127	2.32	0.95	0.35	0.17	Q157	7.61	0.93	0.63	0.20
Q68	-7.38	1.14	0.23	0.48	Q98	-1.66	0.50	0.84	0.25	Q128	3.82	0.42	0.15	0.02	Q158	8.63	0.99	0.49	0.20
Q69	-8.76	1.12	0.15	0.37	Q99	-1.19	1.21	0.62	0.38	Q129	3.74	0.39	0.15	0.02	Q159	7.81	0.86	0.61	0.20
Q70	-4.33	1.02	0.23	0.28	Q100	-2.58	0.92	0.76	0.30	Q130	3.32	1.07	0.17	0.04	Q160	8.51	0.87	0.51	0.19
Q71	-5.29	1.25	0.55	0.42	Q101	-1.93	0.93	0.83	0.26	Q131	3.67	0.74	0.26	0.03	Q161	8.10	1.07	0.56	0.22
Q72	-5.89	0.72	0.75	0.32	Q102	-1.11	1.26	0.72	0.33	Q132	4.40	0.93	0.21	0.03	Q162	7.43	0.85	0.66	0.19
Q73	-6.32	1.15	0.54	0.41	Q103	-1.61	1.21	0.77	0.30	Q133	4.40	1.24	0.20	0.04	Q163	8.80	1.31	0.44	0.23
Q74	-5.96	0.93	0.40	0.31	Q104	-1.19	1.26	0.73	0.33	Q134	5.40	1.23	0.14	0.03	Q164	10.44	0.91	0.26	0.12
Q75	-4.02	1.08	0.29	0.48	Q105	-1.25	1.17	0.75	0.31	Q135	5.92	0.74	0.12	0.04	Q165	6.05	0.97	0.78	0.17
Q76	-5.46	0.94	0.39	0.35	Q106	-1.62	0.98	0.82	0.27	Q136	6.06	0.65	0.19	0.04	Q166	9.05	1.14	0.42	0.20
Q77	-5.35	1.01	0.31	0.38	Q107	-1.06	0.89	0.78	0.28	Q137	6.06	0.62	0.19	0.04	Q167	8.09	0.91	0.57	0.20
Q78	-4.71	1.03	0.31	0.43	Q108	-0.08	0.82	0.68	0.28	Q138	6.94	0.75	0.14	0.04	Q168	9.28	1.25	0.37	0.20

Note. θ = item difficulty; Infit = Infit mean-square; Correlation = Point-biserial correlation.

SUPPLEMENTARY RESULTS

Confirmatory analysis of three-way interaction

To better understand the significant interaction effect observed between age, prenatal adversity, and the BDNF ePRS on cognitive development, using median split we stratified the sample into low and high ePRS and low and high prenatal adversity and repeated the analysis. We observed for the high BDNF ePRS group a significant interaction effect between age and prenatal adversity on cognitive development (B = 0.40, p = .001), while for the low ePRS group the interaction effect was not significant (B = 0.01, *P* = .35). Within the high BDNF ePRS group the age effect on cognitive development was significant for both the low and high prenatal adversity group, with the steepest slope for the low adversity group ($B_{\text{low adversity}}$= 10.1, *P* <0.001, $B_{\text{high adversity}}$ = 9.7, *P* <0.001). This suggest that in children with high BDNF ePRS, higher prenatal adversity was associated with smaller cognitive development in comparison with those exposed to lower prenatal adversity.

Parallel independent component analysis

Table S2. *Brain-phenotype component 5 (B5).*

Area	Broadmann area	L/R volume (cc)	L/R random effects: Max Value (x, y, z)
**Negative**			
Middle Frontal Gyrus	6, 9	0.4/0.5	7.6 (-24, 7, 45)/5.5 (36, 29, 32)
Middle Temporal Gyrus	21, 39	0.1/0.6	3.9 (-49, -56, 10)/6.7 (56, -33, -11)
Sub-Gyral	40	0.4/0.3	5.4 (-22, 29, 32)/5.8 (37, 47, 3)
Precuneus	7	0.3/0.1	5.3 (-21, -59, 43)/4.1 (21, -50, 52)
Middle Occipital Gyrus	18	0.3/0.1	5.1 (-36, -73, -9)/3.5 (40, -77, 14)
Superior Temporal Gyrus	13, 42	0.1/0.0	5.0 (-62, -30, 11)/-999.0 (0, 0, 0)
Precentral Gyrus	6	0.1/0.5	3.5 (-45, 2, 44)/5.0 (59, -7, 29)
Lingual Gyrus	18, 19	0.1/0.1	4.7 (-7, -80, -2)/4.9 (21, -64, -3)
Inferior Parietal Lobule	40	0.3/0.2	4.6 (-53, -32, 28)/3.8 (49, -41, 46)
Superior Frontal Gyrus	6, 10	0.2/0.3	3.8 (-24, 44, 27)/4.6 (34, 49, 14)
Inferior Occipital Gyrus	18	0.1/0.0	4.5 (-24, -88, -9)/-999.0 (0, 0, 0)
Cingulate Gyrus	31, 32	0.1/0.1	3.6 (-7, -38, 36)/4.2 (21, 8, 47)
Cuneus	18, 19	0.1/0.3	3.8 (-15, -95, 13)/4.1 (10, -80, 34)
Superior Parietal Lobule	7	0.1/0.1	3.9 (-24, -45, 59)/4.1 (24, -52, 61)
**Positive**			
Middle Temporal Gyrus	21	0.5/0.9	6.4 (-55, -40, -9)/7.7 (40, -66, 16)
Sub-Gyral	6, 40	0.5/1.0	6.0 (-43, -51, -6)/6.9 (34, -30, 40)
Inferior Temporal Gyrus	19, 20	0.3/0.4	5.0 (-59, -17, -18)/6.5 (52, -13, -21)
Middle Occipital Gyrus	19	0.2/0.4	4.4 (-45, -77, 9)/5.6 (40, -74, 0)
Lingual Gyrus	17, 18, 19	0.3/0.4	5.5 (-15, -60, 0)/4.4 (19, -73, -5)
Fusiform Gyrus	37	0.3/0.1	5.2 (-50, -10, -25)/5.3 (48, -36, -15)
Middle Frontal Gyrus	8, 10	0.4/0.7	5.2 (-37, 26, 30)/4.8 (25, 49, 13)
Inferior Parietal Lobule	40	0.3/0.9	4.3 (-55, -42, 38)/5.1 (43, -50, 45)
Cingulate Gyrus	24	0.0/0.1	-999.0 (0, 0, 0)/4.5 (9, 25, 37)
Precentral Gyrus	6	0.1/0.1	3.5 (-36, 22, 35)/4.5 (49, -17, 34)
Postcentral Gyrus	5	0.2/0.1	3.8 (-52, -18, 23)/4.4 (36, -27, 43)
Precuneus	7	0.1/0.3	4.2 (-36, -73, 35)/4.3 (16, -47, 56)
Cuneus	19	0.1/0.1	3.8 (-12, -81, 36)/4.1 (12, -93, 10)
Angular Gyrus	39	0.1/0.1	4.0 (-36, -76, 30)/3.7 (33, -58, 37)
Posterior Cingulate	30	0.0/0.1	-999.0 (0, 0, 0)/4.0 (19, -64, 10)
Superior Parietal Lobule	7	0.1/0.1	3.8 (-39, -57, 48)/4.0 (30, -57, 47)
Inferior Occipital Gyrus	19	0.1/0.1	3.9 (-40, -77, -4)/3.8 (43, -76, -3)

Table S3. *Brain-phenotype component 6 (B6).*

Area	Broadmann area	L/R volume (cc)	L/R random effects: Max Value (x, y, z)
**Negative**			
Precentral Gyrus	6	0.1/0.2	7.3 (-34, 21, 35)/4.9 (36, 19, 36)
Middle Temporal Gyrus	21	0.5/0.2	6.6 (-40, -69, 20)/4.6 (56, -38, -8)
Middle Frontal Gyrus	8, 9	0.5/0.4	5.4 (-33, 24, 38)/6.6 (34, 26, 30)
Superior Frontal Gyrus	10	0.3/0.4	4.5 (-27, 51, -5)/6.0 (21, 60, 5)
Supramarginal Gyrus	40	0.2/0.1	5.8 (-48, -48, 31)/3.7 (48, -49, 31)
Postcentral Gyrus	1, 3	0.8/0.1	5.6 (-50, -22, 33)/3.8 (58, -24, 39)
Inferior Parietal Lobule	7, 39, 40	0.5/0.3	5.3 (-34, -59, 39)/4.2 (52, -34, 46)
Precuneus	7	0.4/0.7	4.6 (-16, -58, 28)/5.0 (13, -59, 35)
Cuneus	17, 18, 19	0.2/0.7	4.7 (-3, -70, 19)/4.7 (12, -88, 18)
Inferior Temporal Gyrus	19	0.1/0.0	4.4 (-48, -60, -2)/-999.0 (0, 0, 0)
Medial Frontal Gyrus	6	0.1/0.2	4.3 (-19, 7, 49)/4.1 (16, 4, 59)
Inferior Frontal Gyrus	9	0.1/0.1	3.8 (-48, 4, 29)/3.6 (48, 35, 7)
Superior Temporal Gyrus	41	0.1/0.0	3.7 (-42, -34, 17)/-999.0 (0, 0, 0)
Superior Parietal Lobule	7	0.1/0.0	3.6 (-22, -61, 58)/-999.0 (0, 0, 0)
Cingulate Gyrus	24	0.0/0.1	-999.0 (0, 0, 0)/3.6 (4, -17, 37)
**Positive**			
Anterior Cingulate	32	0.1/0.0	3.9 (-6, 36, -8)/-999.0 (0, 0, 0)
Cingulate Gyrus	31	0.1/0.1	3.6 (-7, -40, 37)/3.9 (7, -40, 41)
Cuneus	18, 19, 30	0.1/0.1	3.5 (-7, -86, 22)/3.8 (19, -88, 24)
Fusiform Gyrus	20	0.1/0.1	4.6 (-52, -10, -23)/5.6 (55, -19, -24)
Inferior Frontal Gyrus	9, 47	0.4/0.4	4.9 (-46, 32, -8)/5.8 (52, 6, 31)
Inferior Occipital Gyrus	18	0.1/0.1	3.9 (-31, -86, -6)/4.4 (37, -70, -4)
Inferior Parietal Lobule	40	0.4/0.1	4.4 (-50, -34, 45)/5.4 (53, -31, 40)
Inferior Temporal Gyrus	20, 21	0.3/0.2	5.2 (-53, -21, -18)/3.9 (58, -24, -19)
Lingual Gyrus	18	0.3/0.1	4.2 (-27, -70, -5)/5.3 (21, -80, -2)
Medial Frontal Gyrus	10	0.1/0.2	3.7 (-7, 60, -4)/4.4 (9, 57, 0)
Middle Frontal Gyrus	6, 8, 10	0.9/1.0	5.4 (-31, 27, 37)/6.3 (25, 19, 39)
Middle Occipital Gyrus	19	0.1/0.1	5.6 (-40, -79, 3)/4.3 (45, -74, 9)
Middle Temporal Gyrus	21	0.8/0.5	5.8 (-61, -28, -11)/5.6 (42, -72, 23)
Parahippocampal Gyrus	36	0.0/0.1	-999.0 (0, 0, 0)/3.8 (36, -20, -24)
Postcentral Gyrus	2, 4, 40	0.4/0.2	4.0 (-34, -31, 50)/4.3 (13, -36, 58)
Precentral Gyrus	4	0.1/0.0	3.6 (-55, -14, 39)/-999.0 (0, 0, 0)
Precuneus	7, 19	0.6/0.7	5.2 (-24, -64, 35)/7.1 (19, -57, 47)
Superior Frontal Gyrus	6, 9	0.4/0.2	5.3 (-24, 49, 9)/4.4 (13, 47, 31)
Superior Temporal Gyrus	22, 39, 42	0.3/0.0	5.8 (-59, -10, 1)/-999.0 (0, 0, 0)

Table S4. *Brain-phenotype component 8 (B8).*

Area	Broadmann area	L/R volume (cc)	L/R random effects: Max Value (x, y, z)
**Negative**			
Precuneus	7, 19, 31	0.6/0.5	5.2 (-7, -56, 50)/7.3 (6, -54, 32)
Middle Frontal Gyrus	10, 11	0.6/0.8	6.7 (-30, 33, 26)/4.8 (36, 50, -11)
Middle Temporal Gyrus	21, 39	0.8/0.4	5.2 (-61, -27, -8)/6.5 (52, -48, -1)
Precentral Gyrus	4	0.0/0.3	-999.0 (0, 0, 0)/6.1 (34, -28, 53)
Inferior Frontal Gyrus	9, 45, 47	0.6/0.6	5.4 (-49, 23, 3)/5.4 (21, 15, -17)
Middle Occipital Gyrus	18	0.2/0.4	5.0 (-27, -78, 20)/5.4 (37, -77, 4)
Superior Frontal Gyrus	9, 10, 11	0.6/0.5	5.4 (-13, 61, -7)/5.1 (19, 49, 21)
Cuneus	17, 18, 19, 23	0.3/0.6	5.1 (-13, -91, 24)/4.5 (18, -79, 26)
Superior Parietal Lobule	7	0.1/0.1	3.8 (-9, -63, 54)/5.0 (28, -56, 44)
Inferior Parietal Lobule	40	0.2/0.2	4.6 (-43, -61, 39)/4.7 (39, -56, 46)
Inferior Temporal Gyrus	20	0.0/0.3	-999.0 (0, 0, 0)/4.5 (46, -71, 1)
Insula	13	0.1/0.0	4.4 (-40, 15, 6)/-999.0 (0, 0, 0)
Lingual Gyrus	18, 19	0.4/0.1	4.1 (-25, -72, -3)/4.3 (19, -88, -6)
Anterior Cingulate	32	0.1/0.1	3.7 (-4, 42, -5)/4.2 (10, 39, -1)
Medial Frontal Gyrus	10	0.3/0.3	4.1 (-7, 37, -11)/4.1 (9, 35, 30)
Superior Temporal Gyrus	22	0.1/0.1	4.1 (-58, -52, 10)/3.6 (46, -46, 19)
Inferior Occipital Gyrus	18	0.0/0.1	-999.0 (0, 0, 0)/3.8 (28, -85, -7)
Fusiform Gyrus	20	0.1/0.0	3.7 (-34, -53, -10)/-999.0 (0, 0, 0)
**Positive**			
Supramarginal Gyrus	40	0.3/0.1	7.9 (-48, -46, 34)/4.4 (56, -35, 36)
Inferior Parietal Lobule	40	0.8/0.2	6.8 (-48, -46, 38)/4.8 (59, -33, 33)
Sub-Gyral	6, 8, 40	0.6/0.3	6.7 (-24, 31, 30)/5.8 (27, -56, 54)
Superior Temporal Gyrus	39	0.1/0.6	4.9 (-40, -53, 23)/6.0 (42, -54, 18)
Middle Temporal Gyrus	39	0.4/0.3	5.4 (-56, -40, -8)/4.6 (42, -60, 15)
Superior Frontal Gyrus	6, 10	0.4/0.5	4.5 (-16, 53, 19)/5.3 (22, 50, 13)
Fusiform Gyrus	20, 37	0.2/0.1	4.2 (-48, -9, -25)/5.2 (42, -30, -15)
Inferior Temporal Gyrus	20, 37	0.3/0.3	5.1 (-50, -26, -20)/5.1 (55, -17, -21)
Precentral Gyrus	4, 6	0.2/0.2	4.2 (-59, -10, 27)/5.1 (58, 1, 12)
Lingual Gyrus	18, 19	0.2/0.3	4.7 (-16, -78, -7)/5.0 (9, -70, 3)
Middle Frontal Gyrus	6, 8, 9	0.7/0.4	5.0 (-34, 50, 3)/4.6 (46, 32, 22)
Inferior Frontal Gyrus	13	0.3/0.1	4.6 (-34, 7, 33)/4.6 (49, 32, 7)
Medial Frontal Gyrus	6, 10	0.3/0.3	4.6 (-9, 49, 17)/4.5 (15, 4, 59)
Middle Occipital Gyrus	18, 19	0.5/0.4	4.6 (-39, -70, -8)/4.2 (31, -84, 0)
Anterior Cingulate	32	0.1/0.0	4.6 (-6, 36, 13)/-999.0 (0, 0, 0)
Precuneus	31, 39	0.3/0.3	4.4 (-7, -51, 36)/4.5 (9, -57, 28)
Inferior Occipital Gyrus	18	0.1/0.0	4.4 (-33, -78, -6)/-999.0 (0, 0, 0)
Superior Parietal Lobule	7	0.1/0.1	3.7 (-15, -63, 53)/4.1 (30, -54, 61)

Table S5. *Descriptive statistics of genetic and brain Independent Component loading coefficients*

IC	High prenatal adversity		Low prenatal adversity		*t*	*P*
	Mean	*SD*	*Mean*	*SD*		
G1	-0.01	0.01	-0.01	0.01	-0.19	0.85
G2	0.00	0.01	0.00	0.01	1.17	0.25
G3	0.01	0.02	0.00	0.01	2.05	0.05
**G4**	**-0.01**	**0.01**	**-0.01**	**0.01**	**-2.08**	**0.04**
G5	-0.01	0.01	-0.01	0.01	0.39	0.70
G6	0.00	0.01	0.01	0.01	-1.84	0.07
G7	0.01	0.01	0.01	0.01	0.12	0.90
G8	-0.01	0.01	-0.01	0.01	-0.36	0.72
G9	-0.08	0.01	-0.07	0.01	-1.63	0.11
**G10**	**-0.03**	**0.01**	**-0.04**	**0.01**	**2.38**	**0.02**
**G11**	**-0.02**	**0.01**	**-0.01**	**0.01**	**-2.07**	**0.04**
G12	0.00	0.01	0.00	0.01	1.27	0.21
G13	0.00	0.01	0.00	0.01	-1.69	0.10
G14	-0.01	0.01	-0.01	0.01	0.70	0.48
G15	0.01	0.01	0.01	0.01	-0.22	0.83
B1	0.22	0.02	0.23	0.02	-1.84	0.07
B2	0.01	0.03	0.00	0.03	0.44	0.66
B3	0.05	0.05	0.06	0.06	-0.56	0.58
**B4**	**0.00**	**0.02**	**0.02**	**0.03**	**-2.16**	**0.04**
**B5**	**0.02**	**0.03**	**0.01**	**0.03**	**2.11**	**0.04**
B6	0.01	0.03	0.02	0.03	-1.71	0.10
**B7**	**0.02**	**0.03**	**0.00**	**0.03**	**2.97**	**<0.01**
**B8**	**-0.01**	**0.03**	**0.01**	**0.02**	**-3.33**	**<0.01**

Note. Student's t-test was applied to compare mean loading coefficients between low and high prenatal adversity groups.
